# Evaluating human–machine collaboration through a comparative analysis of experts, machine learning, and hybrid approaches in real estate valuation

**DOI:** 10.1038/s41598-025-34099-9

**Published:** 2026-01-17

**Authors:** Christopher Kmen, Gerhard Navratil, Markus Kattenbeck, Ioannis Giannopoulos

**Affiliations:** 1 Geoinformation Group, TU Wien, 1040 Vienna, Austria; 2 OTTO Immobilien GmbH, Research, 1010 Vienna, Austria

**Keywords:** Human subject study, Expert assistance, Real estate price prediction, Transaction data, machine learning algorithms, Model evaluation, Engineering, Mathematics and computing

## Abstract

Accurate prediction of real estate prices remains a major challenge due to dynamic market conditions and the limitations of traditional valuation methods. Empirical studies that directly compare human experts, machine learning (ML) models, and hybrid approaches are rare. This study examines the predictive accuracy and efficiency of an XGBoost-based ML model, real estate experts, and a hybrid human–machine approach. A model was trained using 21,736 real estate transactions from Vienna (2018–2022). We then conducted an experimental procedure with 13 experts who evaluated newly built apartments sold in 2023 under three conditions: limited information, state-of-the-art expert methods, and collaboration between experts and ML model. The results show that the ML model achieves accuracy comparable to that of experts while significantly reducing the time required for the task. Within the hybrid approach, experts were able to achieve the highest accuracy in comparison to other methods. These results underscore the potential of human-ML collaboration.

## Introduction

The real estate market is constantly evolving and has undergone significant transformations due to local and global economic booms and recessions in the past two decades^1,2^. Real estate serves a dual purpose: it fulfills a basic human need for housing and represents a major form of investment. Consequently, this emotionally charged topic garners extensive coverage in both the media and academic research. Therefore, ongoing research is essential to understand the dynamics of this rapidly evolving market and develop tools that can more accurately predict current price levels and future trends.

The use of models to support decision-making in the real estate market has a long history, dating back decades and, depending on perspective, even centuries. Early examples include François Quesnay’s “Tableau Économique” (1758)^3^ and the Habsburg Empire’s Josephinian Cadastre (1785)^4^, which laid the groundwork for modern property valuation research. The foundational work for modern real estate estimation models began in the mid-1970s with the development of the hedonic price evaluation by^5^, setting the stage for subsequent advancements in modeling and methodology. Over the past few decades, these models have been refined, leading to various approaches aimed at assessing and forecasting property values. Hedonic price models (HPMs) are particularly notable for their practical applications and theoretical foundations. Widely used by real estate professionals, HPMs help in assessing property values and establishing benchmarks for comparison. These models often operate as standalone tools or supplementary aids, allowing experts to accurately value properties^6^.

With the rise of machine learning and deep learning, advanced techniques have been applied to the prediction of property prices. Modern approaches have introduced a variety of new models, some integrating with HPMs^7,8^, while others demonstrate that contemporary machine learning techniques better adapt to the increasingly dynamic market and urban landscapes^9^. A wide range of modeling approaches have been explored using diverse algorithms, with tasks varying significantly based on available data. Models that work with transactional data, considered the most reliable, are limited by data availability^10^. Although these models can demonstrate the feasibility of different modeling architectures and methodologies, their associated errors remain more theoretical than practical, due to the restriction to historical data. This raises important questions about the real-world effectiveness of these models and how they can be practically tested.

There is a growing interest in understanding how humans and AI can effectively collaborate in applied decision-making domains such as real estate valuation. Since data can only be collected in a historical context, the challenge is to utilize these data to accurately predict future trends and verify these results. Furthermore, it is essential to validate these predictions with unseen data and to compare how a predictive model performs against experts in this market. A comparison with experts would provide a valuable link to real-world applications. Using an updated version of the price prediction model presented in^11^, our research bridges the gap between theoretical and practical approaches. To assess how well our model performs in real-world scenarios, we focus on the following key questions:

1. How can a real-world scenario be created and tested?
2. How effectively does the model perform compared to human experts when both have the same foundational information about a property?
3. How does the model compare when human experts employ standard evaluation methods?
4. Do human experts benefit from collaborating with our model?

These questions are explored through a within-subject design study involving 13 experts. These experiments not only assess price prediction accuracy, but also examine whether machine learning models can decrease the time required to complete tasks and reduce associated stress.

## Related work

A review of research on AI-assisted decision-making systems reveals three major strands relevant to designing effective solutions for complex tasks, such as real estate price prediction. The first strand focuses on human–AI collaboration, where AI enhances human decision making.^12^ emphasize the importance of transparent AI systems and a clear composition of the output to avoid overreliance or distrust.^13^ also highlight the risks of negative biases, such as overreliance or skepticism, stressing the need to balance human autonomy with AI support.^14^ further suggest that interpretability of AI systems is crucial for fostering trust and improving collaborative decision-making.^15^ add that domain experts tend to selectively follow AI recommendations depending on how well they perceive the system to be consistent with their knowledge and task context. Furthermore, research shows that transparency alone does not automatically increase trust; instead, the perceived source and interpretability of AI results play a decisive role in whether users accept or reject automated suggestions^16,17^.

The second strand focuses on error mitigation and bias management.^18^ discusses how the combination of human intuition with automation can reduce errors and improve decision accuracy in complex environments. ^19^ add that allowing users to interact with and adjust AI outputs builds trust and engagement, although caution is necessary to prevent overreliance.^20^ stress that trust repair is vital after system errors, recommending actions such as apologies or explanations to rebuild user confidence in human–AI collaboration, particularly after failures.

The third strand examines the balance between human expertise and AI predictions.^21^ propose that hybrid intelligence systems, combining human intuition with AI capabilities, achieve superior results leveraging the strengths of both: AI efficiently processes large datasets, while human expertise remains essential for interpreting ambiguous cases.^22^ echo this, noting that human judgment is crucial even when algorithmic insights are available, particularly in nuanced decision making.^23^ adds that explainable AI systems help bridge the gap between human understanding and machine output, reducing errors, and fostering better collaboration by aligning AI decisions with human reasoning.

In conclusion, these strands provide critical insights for designing expert experiments with AI-assisted decision systems. Balancing human autonomy and AI support is essential to prevent overreliance or skepticism, especially when experts collaborate with AI. Transparent and well-explained AI outputs foster trust and engagement, while allowing experts to adjust predictions mitigates errors. Understanding how AI improves or complements expert judgment is crucial for testing hybrid systems in complex decision making, such as real estate pricing.

### Experimental landscape

While these conceptual frameworks guide the design of human–AI collaboration systems, it is equally important to examine prior empirical research in domains closer to real estate valuation to contextualize the present study. In this regard, examining how human judgment and behavioral factors influence real estate price formation provides a relevant empirical parallel. Research on human factors in the formation of real estate prices is limited, yet it has been a subject of study for some time. In particular, a study conducted in the mid-1980s explored the impact of the anchor effect on property valuation across different types of participants. In the work by^24^, participants, including real estate agents and undergraduates, received detailed property information along with a list price that served as an anchor. They then estimated the property’s value, allowing researchers to examine how the initial anchor influenced their final valuation. This study highlighted the significant role of the anchoring effect in pricing decisions. More recently,^25^ showed that anchoring remains a persistent cognitive bias even in business intelligence environments, despite the availability of extensive information to support decision-making.

A recent study by^26^ proposes an automated valuation model for the residential real estate market using stacked generalization and comparable market analysis. The goal is to assess the predictive power and accuracy of machine learning methods compared to human real estate agents. However, this study is observational rather than experimental, as it relies on pre-existing agent price data rather than controlled testing. The agent prices used for comparison are collected from Finn.no, a popular Norwegian real estate website, and represent agents’ price estimates (offer prices).

^27^ explore the interpretability of machine learning models to aid human decision making. Their study focuses on understanding whether interpretable models achieve their intended effects. The authors conducted a series of experiments with 3800 participants recruited through Amazon Mechanical Turk. Participants predicted the prices of New York apartments, assisted by machine learning models. The experiment had a 2 $\times$ 2 design where participants saw either a two- or eight-feature model presented as clear or black box. Each participant was shown the same set of apartments and asked to predict prices, first without and then with model assistance. The study measured how well participants could simulate the model’s predictions, follow beneficial predictions, and detect significant model errors. The findings revealed that participants could better simulate clear models with few features, but did not follow the predictions more closely and were less able to detect significant model errors due to information overload.

^28^ examine the impact of predictive uncertainty on decision making in machine learning-assisted contexts. The study involved 190 participants who predicted monthly rental prices of Cambridge, MA apartments, using various types of uncertainty distribution to model uncertainty. Participants were shown different types of distribution. The study found that displaying uncertainty generally led to smaller disagreements with predictions, especially among participants familiar with machine learning. This suggests that predictive uncertainty can be a useful decision aid, but its impact varies based on the type of distribution and expertise of the user.

Although comparing machine-created output against humans has seen limited research in the real estate niche, there are many other fields where this is done more frequently. ^29^ investigated optimal collaboration between human radiologists and AI in diagnostic settings, highlighting the potential and limitations of AI assistance. Their findings suggest that, while AI predictions can outperform most human experts, overall diagnostic performance does not always improve when humans are assisted by AI due to errors in belief updating. This study underscores the importance of considering both AI predictions and contextual information provided by human experts to achieve optimal outcomes. They conducted an experiment with 227 radiologists working in four different information environments: X-ray only (XO), X-ray with AI predictions (AI), X-ray with clinical history (CH) and X-ray with AI predictions and clinical history (AI+CH). The precision of their assessments was evaluated against a diagnostic standard. The study used randomized control trials to ensure a varied exposure to the different environments and treatment effects were analyzed based on deviations from the diagnostic standard and the time taken per case. This paper is particularly valuable, as it offers a comprehensive approach to comparing various input combinations, both with and without AI, to better understand their impacts. This holistic methodology addresses a gap in real estate experimental design, which often lacks such in-depth comparisons.

The large body of research on human interaction with AI in various fields, as highlighted by^30^, emphasizes the growing interest in improving human decision making with AI assistance. This meta-study surveys over 100 studies, providing insight into decision tasks, AI models and evaluation metrics across domains. The findings emphasize the need for empirical approaches to understand human-AI interaction and develop effective systems. Many studies evaluate the impact of AI on decision-making, focusing on trust, reliance, and performance. These studies often show AI significantly improves decision outcomes, especially in complex tasks where human expertise alone may be insufficient. Despite limited research in real estate, there is significant potential to enhance real estate prediction models.

### Experimental design critic

While these studies offer valuable methodological insights, several critical limitations remain in existing real estate research, particularly regarding validation practices and experimental realism. One key critique focuses on the validation of the results and the methods used for comparison. For example,^26^ is based on offer prices collected from a real estate website. However, as^11^ have pointed out, offer prices can differ significantly from actual transaction prices, creating a potential disconnect between model predictions and true market values.

There is a growing interest in research on human interaction with artificial intelligence, as highlighted by the recent meta-study of^30^. This work emphasizes that many studies fail to align with real-world applications, particularly when investigating high-stakes domains without appropriate study design—including suitable participants, realistic consequences, and contextual framing—which can lead to misaligned conclusions. Furthermore, tasks defined based on readily available datasets may not reflect realistic decision-making scenarios. This gap is particularly evident in the real estate sector, where empirical studies on human–AI collaboration remain scarce. This scarcity is due not only to the lack of up-to-date data but also to the disconnect between theoretical research and industry practice. Using actual transaction data, we designed an empirical study comparing predictions from human experts, machine learning models, and hybrid approaches (experts supported by ML-based predictions).

## Methods

This section outlines the experimental design. It first introduces the conceptual framework, describing the rationale, objectives, and three experimental conditions developed to evaluate the comparative and collaborative performance of human experts and a machine learning model in real estate price prediction. The subsection ’Human Subject Experiment’ describes the experimental setup in detail, including selected data points, participants, materials, and procedures. In addition, the design challenges and the methodological safeguards implemented to ensure realism and minimize bias are presented. Finally, the evaluation metrics used are specified, such as accuracy, effort, and time efficiency, and their application under experimental conditions is explained.

### Concept

The literature review provides several key insights that inform the experimental design. The anchoring effect significantly impacts real estate price estimates, while machine learning models show potential but struggle with discrepancies between offer and transaction prices. Research emphasizes the importance of model transparency and uncertainty in enhancing the collaboration between humans and AI. However, there is a notable lack of empirical studies that apply AI-human collaboration specifically to real estate pricing using real transaction data, highlighting the need for experiments like those conducted in this study.

To address the lack of real-world scenarios in machine learning research involving human interaction, a series of experiments were designed. This study aims to evaluate the effectiveness and accuracy of the XGBoost^31^ model, introduced by^11^ to predict real estate prices under various conditions. The performance of the model will be compared with that of human experts to ensure a comprehensive evaluation. The choice of human experts for the experiments is based on their professional background, with several professions deemed suitable. Real estate agents are included because their daily business involves estimating reasonable selling prices or verifying whether an offer price is appropriate. Real estate appraisers are chosen because their job is to determine the value of a property. Finally, investment agents who handle transactions in apartment complexes are considered, as they must have knowledge about square meter prices of apartments to accurately price whole complexes.

The research seeks to determine whether the algorithm surpasses human experience and conventional methods in real estate price prediction and if collaboration between the model and human experts can lead to better results. In addition, it explores whether changes in accuracy, time and stress levels can be detected when experts use the model as a support tool, reflecting a hybrid approach. This investigation is motivated by the growing reliance on technology in data analysis in the real estate industry and its potential to improve decision making in real estate valuation.

The experimental design examines several dimensions of human–expert interaction in predictive analytics, structured into three conditions:

1. Condition A: The direct comparison of predictive accuracy between the XGBoost model and human experts under ML-Data parity, where both have access to identical sets of base information for the prediction task.
2. Condition B: The direct comparison of predictive accuracy between the XGBoost model and human predictions when experts use their state-of-the-art approaches to real estate properties.
3. Condition C: The investigation of whether human experts can enhance their predictive accuracy by utilizing the XGBoost model’s outputs combined with their professional judgment and additional contextual information.

Condition A: This condition is designed to assess whether human experts or the machine learning model (XGBoost) performs better when operating under ML-Data parity—meaning both are given access to an identical set of base information. The data is prepared and arranged to be readable for humans. Consequently, no additional resources are allowed for participants in this setup. There are two questions involved in this condition. First, how accurate can an expert be in predicting prices with limited access to features, relying only on open-source data, as it may be the case in real-world scenarios? Second, how much stress and time effort is generated by a task like this? Condition A is designed to be the most controlled, providing participants exclusively with the information used by the predictive model, presented in an accessible format. Although this condition encompasses most characteristics, it does not necessarily provide the most relevant or useful information for an accurate prediction by an expert. The data is presented in structured tables and graphs that are optimized for machine learning rather than human thinking, which limits the ability of experts to examine or contextualize the information. As a result, while the completeness of the features is ensured, interpretability and freedom of information are limited, making effective human evaluation difficult.

Condition B: This condition investigates how well a machine-generated prediction compares to the state-of-the-art methods used by experts. Can a model, which is fed with many years of spatial and socio-economic data, match the performance and experience of professionals involved in price formation? Condition B explores whether experts can outperform the model when they are allowed to employ their state-of-the-art methods to estimate property prices. Under normal circumstances, experts would access professional databases that contain comparable transactions. Since the data used are actual transactions, it would be easy for them to simply look up the transaction in a database. However, from a human subject study perspective, this introduces a bias, as it eliminates uncertainty and artificially reduces the error to zero. Therefore, the comparisons these databases usually provide are shown to the participants. This allows a direct comparison of human expertise with automated predictions under enhanced informational conditions.

Condition C: The final condition evaluates how prediction accuracy changes through collaboration between humans and AI. Participants apply their own valuation methods while also receiving a price estimate from the model. To avoid biases such as overreliance or distrust, participants are told that the estimate comes from a human expert (as discussed by^13^). Participants receive the price predicted by the model and then enter their own revised estimate. The resulting prediction thus reflects a human assessment based on the results of the ML model. Similarly to the previous condition, participants can access external sources, excluding actual transaction data. This setup tests how well experts incorporate machine-generated predictions into their decision making.

In addition to accuracy metrics, the study collected participant demographics and subjective workload ratings to provide a broader view of decision making under different conditions. The experiment also examines the time experts take to complete tasks and their perceived difficulty in the conditions and the usefulness of the information used, based on self-assessments.

### Human subject experiment

Based on this concept, we devised a human-subject experiment with human experts and a pre-trained XGBoost model. This section provides a detailed account of the setup. The first part presents the design, beginning with the selected data points, the model used, the participants, and the conceptual framework. The second part explains the materials presented to the participants and specifies how they were allowed to use additional resources. The final part examines the procedure and is described in detail, explaining how the experiments were conducted.

All methods were conducted in accordance with relevant guidelines and regulations. The experiment protocol, including recruitment, informed consent, and data processing procedures, was reviewed by the Research Ethics Committee of TU Wien (reference number: 048_022024_TUWREC). Written informed consent was obtained from all participants prior to participation. Participation was voluntary and could be discontinued at any time without consequences. No minors were recruited and no identifiable personal data or images are reported. To minimize bias in participant behavior, a temporary, mild form of deception was employed: the model-generated price suggestions were presented as if they originated from a human expert. This measure ensured an unbiased interaction with the information provided and prevented anchoring or automation effects. All procedures were reviewed by the ethics committee and the participants were informed of the purpose of this design decision immediately after each session. No negative reactions or objections were reported during or after the debriefing regarding the deception.

Design: To create a scenario as realistic as possible, transactions from the recent past were selected, specifically data from the second half of 2023. This setup ensures that for the model, it will be a genuine prediction into the future since it only has data up to the end of 2022. For experts, it will be a retrospective analysis, as the experiments are conducted in May 2024. A downside of this scenario is that experts have actual knowledge of prices at the time the data points were sold and even beyond, which might give them an advantage in the prediction task. However, the minimal time gap between the transaction dates and the experiments should ensure that this scenario closely mirrors real-world conditions.

Design challenges: An ideal scenario would involve selecting apartments that have not yet been sold, allowing both the model and the experts to predict their prices. However, this approach is impractical. Data from Exploreal show that an average of 5197 new apartments were built annually between 2021 and 2023, with an average of 3171 newly built apartments sold during this period. Therefore, even selecting properties likely to be sold has a low probability of success. Additionally, since prices are time-sensitive, there is only a finite window during which predictions remain valid. If an apartment is not sold for several months, the prediction process must be repeated due to measurable changes in price. These changes can go in either direction: they may increase due to economic factors such as inflation or decrease as a result of price depreciation caused by overvaluation. Without information on the transaction date, the temporal context of the price prediction remains undefined—a crucial limitation that introduces unavoidable and unquantifiable uncertainty in the final valuation.

XGBoost model: The experiments were built on the model of^11^. XGBoost, a gradient-boosted decision tree algorithm, builds trees sequentially so that each new tree corrects the residual errors of the previous one. In large and heterogeneous datasets, its use of weighted quantile sketching, column subsampling, and L1/L2 regularization helps reduce overfitting while maintaining computational efficiency^31^. XGBoost is well suited for modeling real estate prices in urban environments, as it effectively captures nonlinear and hierarchical interactions between spatial, socioeconomic, and transaction-related features^32–34^. It provides a strong foundation for the present human–machine collaboration experiment because it combines interpretability, computational efficiency, and high predictive performance^35,36^.

Several updates were made to improve the performance and accuracy of the original price prediction model. The data set was expanded to include property data up until 2022, and a new identification system using cadastral records was implemented to reduce duplicate entries and better identify buildings within Austria. Additional data sources were integrated, such as seller and buyer classifications, crime statistics, and the Consumer Price Index (CPI) for economic context. Methodological improvements included recalculating building indices and enhancing connectivity through isochrone-based route calculations, ensuring accurate access to points of interest in Vienna. In addition, we compared the XGBoost with several deep learning architectures, including multi-layer perceptrons (MLP), graph neural networks (GNN), and recurrent neural networks (RNN). In multiple trials using the dataset, XGBoost achieved the lowest prediction error for 1-year forecasts and demonstrated the most stable generalization performance overall. These results confirmed XGBoost as the most robust and interpretable approach for the subsequent human-machine collaboration experiment.

Three model variants, based on input intervals of one, four and five years, were trained using data up to 2021 and tested against data from 2022. The 5-year input model achieved the lowest mean absolute percentage error (MAPE) of 14%. As a result, this variant was trained with the most recent data (2018–2022) and selected for further testing in a human subject experiment. The entire training data set comprised 21,736 real estate transactions for newly built apartments, with 2416 additional transactions used for model validation. The feature space of the model consists of 165 features (Appendix A—Table S1), most of which are spatial in nature, while also incorporating both transactional features (e.g., transaction dates, useable area) and socioeconomic factors (e.g., crime rates, income levels, education) to provide a comprehensive context. The final XGBoost model used the following hyperparameters: max_depth = 14, n_estimators = 800, subsample = 0.8, min_child_weight = 8, learning_rate = 0.01, gamma = 0.2, colsample_bytree = 0.6, and eta = 0.3. We selected these values using randomized search across a predefined parameter space, with 5-fold cross-validation applied on the training set.

Object selection process: The following subsection outlines the process used to select the properties for inclusion in the experiments. The objects of this study consist of real estate transactions of newly constructed or refurbished apartments in Vienna during the second half of 2023. Initially, the available data for 2023 were reviewed, identifying 130 data points that met the preconditions. Each transaction was examined in detail to ensure that no additional investments, such as inventory purchases or external spaces such as cellars, were involved. It was also verified that none of the transactions were performed as investments or involved any subsidies. These filtering steps were necessary to obtain a “clean” price and reduce the variability between the properties for the tasks. To avoid memory-related biases in participants’ judgments, we included exclusively objects in the experimental data set that had not previously been evaluated, listed, or otherwise processed by any of the participating experts. This was ensured by cross-checking internal assignment documents disclosed to the authors.

**Fig. 1:**
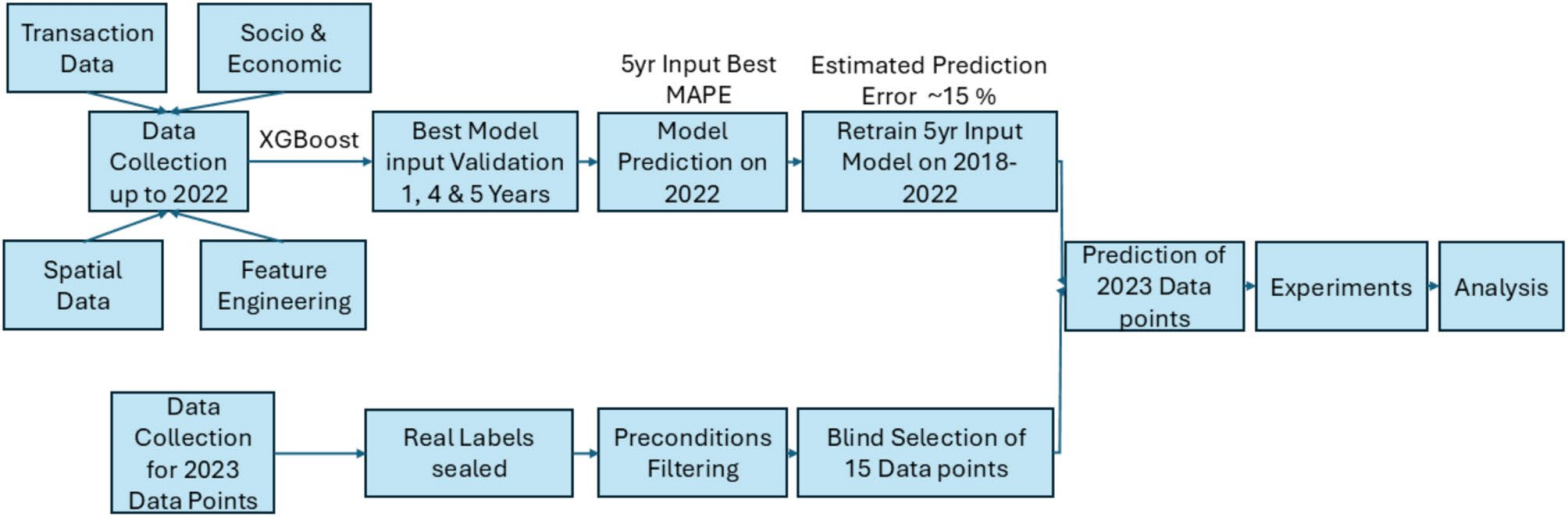
System architecture of the proposed workflow. The diagram illustrates the complete data integration and model development pipeline used for the study. Raw real estate transaction data (2017–2022) was aggregated with spatial and contextual features, including sociodemographic and infrastructure information, and preprocessed for model training. The XGBoost regression model was first trained on data from 2017–2021 and validated on transactions from 2022 to determine the optimal temporal configuration. An input batch of five years yielded the lowest MAPE and was therefore selected for deployment. After validation, the model was retrained using the complete 2018–2022 dataset to generate the final predictions for the human-machine comparison experiment. This architecture ensures temporal consistency between model training, validation, and subsequent expert evaluation.

As a precautionary measure, square meter prices and total prices were separated from the data points to prevent subconscious biases, such as selection bias and confirmation bias, and to ensure that the model was not assigned overly simplistic tasks. By removing these price indicators, the risk that the experimenter unintentionally guides the dataset selection toward expected outcomes is reduced, mitigating the influence of anchoring or availability heuristic. Empirical studies in various fields highlight the necessity of blinding in this context. For example, in clinical trials, the random assignment of participants to treatment groups prevents selection bias and ensures that any observed differences are attributable to the intervention rather than preexisting characteristics^37,38^. Similarly, Ref.^39^ demonstrated that non-blind selection processes can lead to exaggerated effect sizes in patient-reported outcomes, underscoring the importance of blinding in the experimental setup. In the context of surgical trials, Ref.^40^ found that ensuring randomness in selecting participants or procedures for evaluation significantly reduces detection and performance biases, highlighting the universal applicability of such measures. A methodology involving blinded selection of objects aligns with established empirical best practices. This approach minimizes biases such as selection and confirmation bias, protects against cognitive heuristics such as the anchoring effect, and ensures a more objective data selection process. As a result, this approach leads to a more thorough evaluation of the performance of the model in complex real-world situations. Furthermore, to ensure that each transaction was associated with a unique building, transactions within the same building were randomly eliminated, except for one. The final sample included 29 transactions, classified into three geographical subsets (east, middle, west) and three usable area size categories (small, middle, large). From these subsets, five data points were selected for each condition to ensure a diverse representation of the sizes and locations of the properties. Finally, data points were prepared for the experiments. This procedure was chosen to eliminate any possibility of conscious or subconscious influence on the participants during the experiments, as in-situ observation plays a critical role and must be excluded. However, a drawback of this approach is that withholding the prices of the data points could result in outliers within the dataset. Outlier cases were retained in the dataset to preserve the natural variability of real market transactions and to reflect realistic prediction scenarios. Keeping such cases within the dataset may lead to the necessity of excluding them from qualitative analyzes if they are not equally distributed across experimental conditions. The overall system architecture and workflow underlying this study are summarized in Fig. 1, which illustrates the integration of data sources, model development, and experimental setup.

Participants: Thirteen experts participated in the experiments, classified into two groups based on their profession. The first and larger group consists of real estate appraisers, the second group of agents. The disparity between the two groups is due to the availability of experts within the company. In addition, experts are classified into two levels of seniority: juniors with less than five years of experience and seniors with more than five years of experience. The sample includes 6 agents (1 junior, 5 seniors) and 7 appraisers (3 juniors, 4 seniors).

The junior experts had an average of 1.75 years of experience, while senior experts averaged 15.1 years. Regarding market knowledge, 46% rated their familiarity with the Viennese apartment market as Excellent, 39% as Good, and 15% as OK. When asked about the accuracy of their predictions of the correct price per square meter for newly built apartments, only one participant gave himself an excellent rating, while the rest were split between good and OK.

Since the study design required the participation of certified assessment experts, the cohort size is inevitably small. As noted in a recent paper on human–AI collaboration^30^, ecologically valid expert studies structurally limit recruitment because the tasks are time-consuming and the pool of qualified participants is inherently limited. These conditions also apply here: the focus is on professional expertise and realistic tasks rather than statistical scalability. Given such fixed and specialized samples, classical inferential statistical methods are not methodologically appropriate. Consequently, the study is designed as a descriptive and exploratory investigation of expert behavior and the interaction between model and human under realistic working conditions–an established approach in research with small cohorts of human subjects^41–44^.

Materials: The experiments were implemented using a LimeSurvey-based questionnaire (the design of the experiment and the questionnaires were reviewed by the Research Ethics Committee TUW REC), eliminating the need for materials other than a computer. The participants used their office computers, which was crucial because two of the three conditions required access to additional resources to simulate a typical work environment. These resources could include specialized tools and materials specific to the company network or the computer itself.

The LimeSurvey questionnaire was divided into three main sections:

1. Demographic Questionnaire
2. Expertise Questionnaire
3. Experiments

Demographic Questionnaire: The demographic part consists of six personal questions, each varying in form. Participants were asked about their age, biological gender, highest level of education, and current employment status. Additionally, two questions focused on their connection to Vienna: whether they currently reside in Vienna or have lived there in the past, and how many years they have lived in Vienna. The last two questions were particularly important to ensure familiarity with the city, given that participants might not necessarily be from Vienna.

Expertise Questionnaire: The expertise part consists of nine personal questions. Participants were asked if they currently work in the property industry or have ever worked in this industry, and whether they have worked as a real estate agent or property appraiser. Additional questions focused on their current role, asking if they are currently working as a real estate agent or property appraiser and how long they have been in this profession. They were also inquired about any particular types of property or markets they specialize in, and their familiarity with the Viennese property market. Furthermore, participants were asked about their experience in markets other than the Viennese market, the areas of the property industry in which they consider themselves experts, and their self-assessed competence in predicting the purchase price of a new-build apartment in Vienna.

Experiments: Each of the three conditions offers different sets of information to the participants but follows the same structure. The study followed a within-subject design: all participants completed all three conditions (A, B, and C), each consisting of five different and non-overlapping tasks. Participants worked on five tasks, followed by an evaluation of the provided information or, if allowed, self-provided information. All participants assessed the same set of 15 properties under each condition, ensuring comparability between experts and allowing aggregation of individual results into average performance metrics. Each condition is concluded with a NASA Task Load Index (TLX)^45^ questionnaire to capture the effort and stress created by the conditions. The goal for each task in the conditions remains the same: Estimate the transaction square meter price of the given apartment at that point in time. Based on data collection, certain details are consistent across all objects in the various tasks: the sale occurred in the second half of 2023, it is a first-time occupancy, the apartments are not investor properties, there were no subsidies applied, the sale price excludes inventory and additional areas such as car parks, and the seller is a property developer. The core information for each object is consistently presented, and each task includes the ZIP code, purchase date, buyer class, usable floor space, usable area classification, and penthouse status.

The information provided for each condition is described in Table 1.

**Table 1:** Detailed overview of experimental conditions: information provided and tools allowed across different scenarios.

Conditions	Information provided	Tools allowed
Condition A	Object, neighborhood, economy, socio-demographics,	No additional tools allowed
	Education level, infrastructure	
Condition B	Object, completion date, google map link,	Additional tools allowed
	Comparison transactions, $\hbox {m}^2$-Time series district	Except databases showing sales prices
Condition C	As Condition B	As Condition B
	+ Estimated $\hbox {m}^2$ Price	

For further clarification on the content of the information provided, Table 2 offers an overview, presenting a comprehensive breakdown of the included details.

**Table 2:** Detailed overview of information categories provided: This table describes the data given to participants, including object details, neighborhood attributes, economic factors, and available tools, excluding price databases.

Category	Description
Object information	Includes details such as postal code, date of contract, buyer class, usable area, and whether the unit is an apartment or attic
Neighborhood	Measures proximity to Points of Interest (POIs) such as cafes, educational institutions, police, medical facilities, and public infrastructure, with distances at 150 m, 300 m, and 1 km
Economy	Includes data like the ATX index and Consumer Price Index (CPI) at the time of purchase, as well as six months prior
Socio-demographics	Covers population, income, unemployment, and age data at the postal code level
Education level	Presented as a pie chart showing the highest level of education completed
Infrastructure	Details on built-up area, street attributes, and intersections based on a 100 m $\times$ 67 m grid
Completion date	Date and type of the project (new construction or renovation)
Google Map Link	A link to the property’s address on Google Maps, including the ability to view Street View
Comparison transactions	Transactions from the immediate vicinity of the property from the year 2022
$\hbox {m}^2$-Time series district	A history of $\hbox {m}^2$ prices at the postal code level from 2018–2022 for apartment types
Additional tools allowed	Participants can use any tool except databases that display sales prices (e.g., ImmoU, Exploreal)

**Fig. 2:**
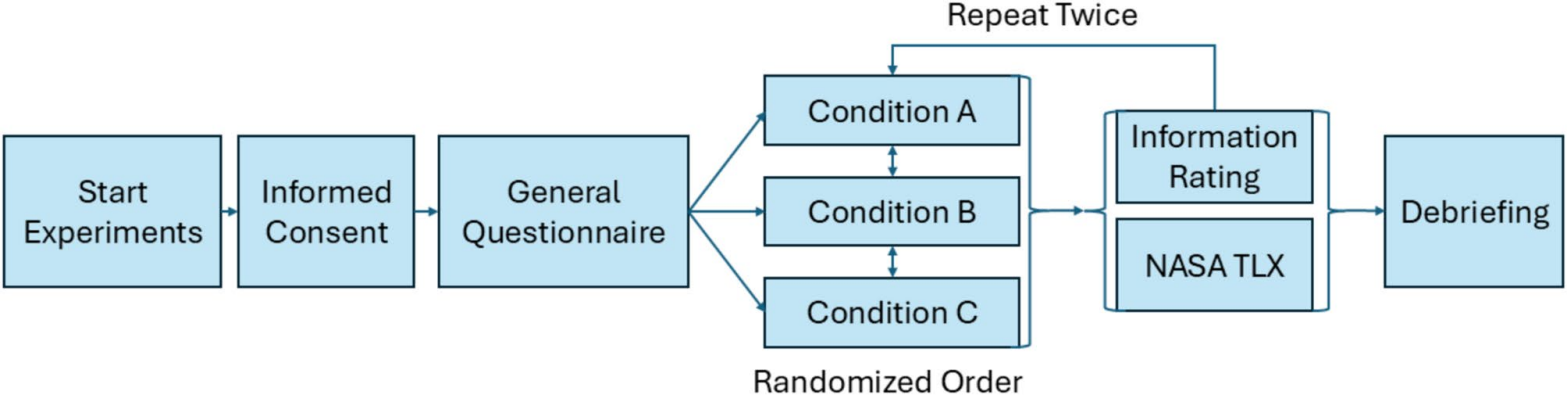
Experimental workflow for the human–AI collaboration study. Thirteen professional appraisers who completed 15 valuation tasks under three randomized conditions: (**A**) Human-only with limited information, (**B**) Expert tools with full access, and (**C**) Hybrid collaboration with model assistance. Each participant predicted the transaction price per square meter, with condition order randomized to mitigate learning effects. Data collected included accuracy (MAPE), task completion time, and subjective workload (NASA TLX). Results were analyzed to compare performance across conditions and evaluate the influence of model support.

As described, after completing the 5 tasks of a condition, an evaluation of the provided information and the self-generated information is carried out. During this evaluation, participants rate each information block from very helpful to not helpful at all. The order of the conditions are randomized to rule out learning effects. The experimental workflow, including the structure of the three conditions, the randomized order of presentation, and the evaluation procedures, is summarized in Fig. 2. A detailed overview of the questionnaire structure is provided in Appendix C (Table S3).

Procedure: The experiments were carried out on multiple occasions, consistently under identical conditions, to ensure standardization. Each session was held in the usual offices of the experts, allowing them access to their computers and specialized tools as permitted by the study protocol. This setup ensured a realistic environment for the experts, allowing them to perform the experiments in the same way they conduct their daily work. However, it also came with some downsides. Due to company office policy, any visual or audio recording was prohibited to address data safety concerns. To maintain the integrity and uniformity of the experimental process, the experimenter provided in situ supervision. Furthermore, this supervision allowed the experimenter to take notes on observations during experiments and debriefing.

Each session began with a brief introduction by the experimenter. After the briefing, the participants were seated at their workstations and provided with a link to the LimeSurvey platform, where the experiments were configured. To maintain control and prevent bias from interaction, participants were required to remain at their workstations and prohibited from conversing with each other throughout the session. Each experiment ended with a brief debriefing to discuss the session and gather immediate feedback from the participants.

A total of four sessions were conducted over 2 days for 13 participants. The relatively small size of this expert group is due to specific requirements that limit eligibility. In addition to needing local expertise and familiarity with the market, experts must also meet legal requirements related to the use of specific tools and databases. In addition, access to these resources requires approval from company executives, including permission for the experimenter to monitor the process. The smallest group consisted of only one person, the largest group of six participants, and the remaining groups consisted of three participants each. Since the experiments were conducted on two separate days one week apart, only minimal additional insight was provided immediately after the experiments to avoid any contradictions. Each group is located in different buildings, spatially separated in various locations. The group examined on the first day was instructed not to discuss the experiments. In addition, direct communication between groups was highly unlikely due to minimal work-related interactions.

A final debriefing was conducted after the completion of all experiments. During this debriefing, participants were informed that they had actually competed against a machine learning model and were given the opportunity to access their results. The complete workflow is presented in Appendix B.

Tasks and measurements: The experimental component of the study is divided into three distinct conditions, each designed to explore different aspects of information processing in decision making. The sequence of conditions is randomized using a Latin square design to mitigate learning biases, and the study employs a within-subject approach. The study participants, all experts, were assigned the following tasks:

1. Human vs. machine with identical information
2. Human vs. machine with additional information for humans
3. Human–machine collaboration

The objective for each task within each condition was consistent: estimate the actual selling price for a given property at a specific point in time.

The following performance metrics were collected during the study:

- Accuracy: Measured using the MAPE for each set of predictions compared to the actual selling prices.
- Task load: Assessed using the NASA TLX, which focuses on mental demand, effort, and frustration experienced by participants during the tasks.
- Information efficiency: The participants rated the importance of each thematic group of information for completing their tasks, with 1 being the most crucial and 5 the least. These thematic groups included categories such as object information, sociodemographics, and others.
- Time efficiency: The time taken by participants to complete each prediction task, comparing the efficiency of human-only versus combined human-machine processes.

## Results and discussion

The results of the experiments are presented in multiple formats. First, a quantitative comparison between experts and the model is presented, focusing on how often participants outperformed the model. Following this, a qualitative analysis examines outliers and individual participant estimates in detail. The frequency of predictions falling within different error thresholds is also reported. This section concludes with an overview of the supplementary information gathered during the experiments.

### Quantitative results

In this first quantitative step, the frequency with which human experts outperformed the model was analyzed. A direct comparison was used to determine which prediction error was smallest in terms of absolute deviation from zero. For each participant, the number of times they outperformed the model based on their MAPE was counted. These counts are then summarized at both the participant and condition levels. The participant-level analysis examines whether improvements can be observed for individual participants. Condition-level analysis provides an overall evaluation of the model. In Conditions A and B, the comparison is straightforward, focusing on whether human predictions were more accurate than those of the model. In Condition C, if humans outperformed the machine, it suggests that the model served as a valid assistance tool.

Table 3 presents the frequencies of participants who outperformed the model in the three conditions. In Condition A, the experts’ predictions were more accurate than those of the model in 37% of cases (24 out of 65). This result was expected, as Condition A is the most challenging for experts due to the limited information provided, which restricts their ability to apply their professional experience effectively.

**Table 3:** Summary of participant performance under conditions A, B, and C. This table shows the number of individual prediction tasks per participant in which their estimate exceeded the model’s prediction. The columns “Total” and “Percentage” indicate how many such cases occurred among all participants under each condition. The best performance of the experts compared to the model was achieved in the hybrid approach in condition C.

Participant ID	Condition A	Condition B	Condition C
1	1	1	3
2	2	2	2
3	2	2	1
4	2	2	5
5	2	4	2
6	4	3	4
7	2	1	5
8	3	3	3
9	2	2	4
10	2	2	0
11	0	3	3
12	1	2	3
13	1	3	1
Sum	24	30	36
Percentage	36.9%	46.2%	55.4%

Condition B is crucial for comparing methods. Experts outperformed the model in 46% of the instances, suggesting that the model meets accuracy standards and reliably generates real-world results. In Condition C, experts outperformed the model 55% of the time, indicating that the model provided effective decision support. Overall, these findings indicate that the hybrid approach enhances performance.

### Qualitative results

Each condition is individually analyzed before comparisons are made. To further contextualize these findings, we now introduce a refined framework for interpreting prediction errors. Although MAPE is an established metric, as defined by^46^, its general classifications are not sufficient to capture the nuances observed in our experiments.

MAPE is traditionally divided into four categories: errors below 10% indicate highly accurate forecasts, errors between 10% and 20% suggest good forecasts, errors between 20% and 50% indicate reasonable forecasts, and errors above 50% reflect inaccurate forecasts. However, these thresholds lack sufficient granularity for detailed evaluation, particularly when analyzing model and expert performance in high-stakes real estate contexts.

To address this, these categories were redefined to better suit the needs of this study. This classification divides MAPE values into the following ranges:

- Errors under 5% MAPE, representing very high accuracy.
- Errors under 10% MAPE, reflecting good accuracy.
- Errors under 15% MAPE, indicating acceptable accuracy.
- Errors above 20% MAPE, signifying poor accuracy.

Condition A represents the scenario of ML-Data parity for experts, in which both human experts and the predictive model have access only to a limited, identical set of information. This condition is particularly challenging for experts, as it requires them to rely heavily on their own knowledge. Serving as a baseline, Condition A provides insight into expert performance under controlled conditions. Table 4 shows considerable variation in expert results. The top of the table shows that the model performed well in four out of five cases, with only the fifth case resulting in a very poor prediction. When comparing the fifth case with the experts’ results, it is evident that their predictions diverged substantially from the model’s. Since the model outperformed experts in four of five cases, the available information was clearly more suitable for the model than for human processing. The model performed with an average error of 10.5%. Only one of the participants was able to achieve a better result than the model. The average expert error was 18.3%, nearly 8% higher than the model. The number of predictions by experts falling into good-accuracy categories ($< 10\%$) was limited, with larger errors ($> 20\%$) occurring frequently, particularly in Task 5.

**Table 4:**
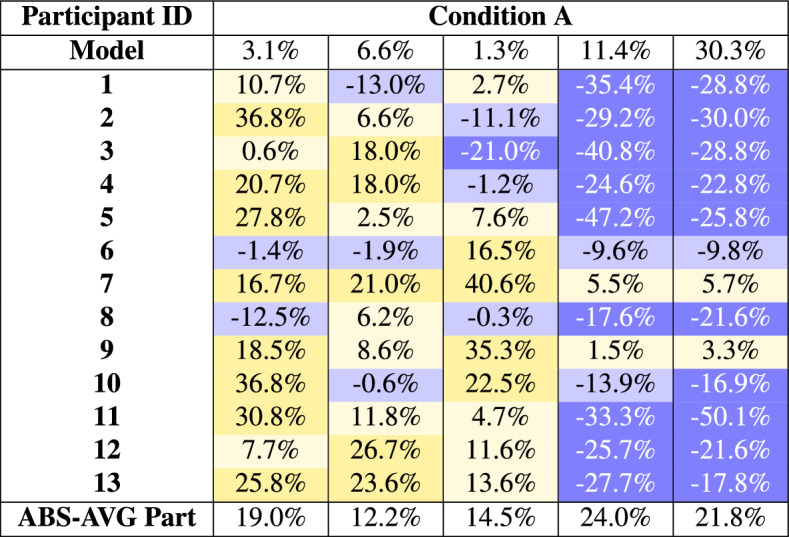
Individual errors of Condition A. Yellow shades represent overvaluation (positive percentages) and blue shades represent undervaluation (negative percentages). The darker the color, the larger the discrepancy from 0%, which is the ideal value.

While the model generally outperformed human experts across the board, some experts demonstrated exceptional accuracy on certain tasks. For example, Expert 9 performed exceptionally well on tasks 4 and 5, with errors of less than 5%. This could indicate a greater familiarity with the districts covered by the task. Other experts struggled with these areas, making 9 and 10 major errors, respectively. Similarly, Expert 6 achieved predictions below 10% MAPE in four out of five tasks, outperforming the model in these cases and demonstrating consistent accuracy.

In general, 43% of the predictions in Condition A fell below the 15% MAPE threshold, suggesting that experts had a reasonable understanding of price structures within districts. However, notable variability in performance was observed, particularly in Task 5, where 10 out of 13 experts made predictions with errors exceeding 15% MAPE. These findings underscore the importance of spatial knowledge and intuition in expert performance under constrained conditions, suggesting that the absence of additional resources limited their ability to achieve precise predictions.

Condition B shown in table 5 serves as the validation condition for the model. In Condition B, the state-of-the-art predictions of the participants and the model itself are compared. The individual results for the experts show a much smaller error rate than in Condition A. Although there are still some outliers in the experts’ predictions, they are much less frequent than in Condition A. Furthermore, the frequency of precise predictions increases significantly. When examining the absolute average error of participants, a substantial decrease is observed, with only one task average exceeding the 15% threshold.

Only one of the model predictions exceeds the 15% threshold and should be considered an outlier. The model performed with an average error of 9.1%. In comparison, the average expert error was 11.7%, and therefore higher than the model. Moreover, the model consistently demonstrated greater accuracy in its predictions compared to the average absolute results of the experts.

**Table 5:**
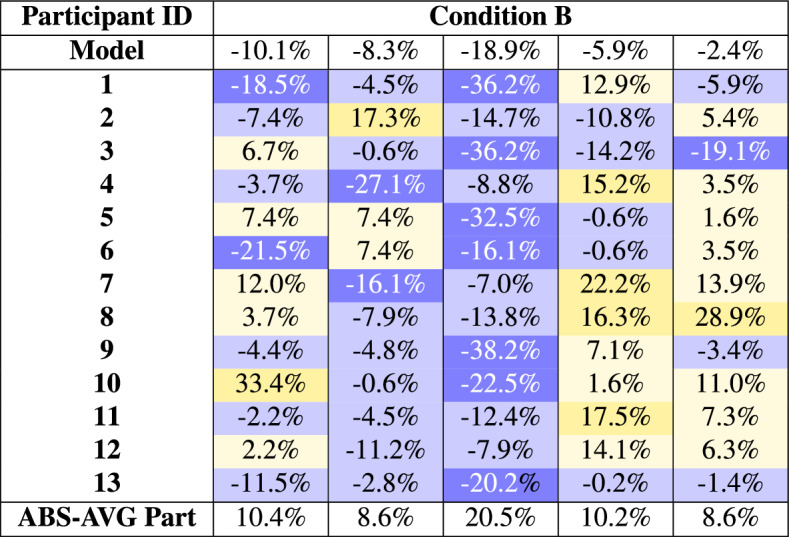
Individual errors of Condition B. Blue shades indicate undervaluation (negative values), and yellow shades indicate overvaluation (positive values). Darker colors represent discrepancies greater than ±15%, with 0% being the ideal outcome.

In Condition B, the number of predictions in the high-accuracy categories ($< 5\%$ and $< 10\%$) increased across tasks, while the occurrence of larger errors ($> 20\%$) decreased. For example, in Task 2, six experts achieved errors under 5%, compared to the best case of three predictions within one task in Condition A. This improvement underscores the effectiveness of enhanced tools in supporting expert decision making.

In general, 70% or more of the predictions in four of five tasks fell below the 15% MAPE threshold, further highlighting the positive impact of state-of-the-art tools. However, variability in performance remained, as multiple experts either underestimated or overestimated the same task, indicating continued discrepancies in expert predictions.

A particularly notable observation in Condition B was Task 3, where the property was an outlier. Both the model and the experts struggled, the model undervaluing the property by almost 19% and all the experts underestimating its price. Despite this, more than half of the experts performed better than the model, with three achieving a MAPE error below 10% and one expert achieving an error as low as 7%. Potential factors contributing to this performance include previous experience with similar properties, familiarity with the location, or the ability to leverage contextual clues more effectively than the model. Task 3 reveals that outliers remain a constant challenge for both models and experts. On average, experts performed slightly worse than the model in Condition B, although the difference was minimal. These findings emphasize that while the model handles general cases effectively, expert intuition and domain knowledge continue to play a critical role, particularly when addressing atypical or outlier properties.

Condition C shown in table 6 represents the hybrid approach of the experiments. A positive indicator of this approach is experts outperforming the model. Two outlier data points were immediately apparent due to poor model predictions. However, the model’s average error of 13.8% is still below the given threshold of 15%.

In this approach, experts were provided with the model’s predictions, making it highly likely that their predictions would be influenced by the model’s output. The experts were informed that the predictions had a possible average error margin of ± 15%. In 10 cases, experts chose to deviate from this given threshold. However, 8 of these deviations led to worse prediction outcomes. Only two of the ten cases resulted in better predictions compared to the model. Both of these successful deviations occurred in the fifth task, where the two experts improved the model’s poor prediction. In contrast, during the first task, where the model also performed poorly, none of the experts deviated from the 15% threshold.

**Table 6:**
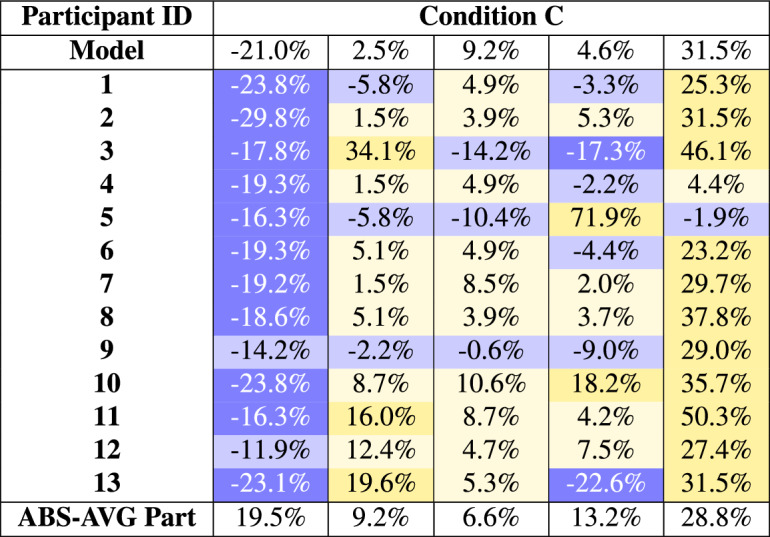
Individual errors of Condition C. Blue shades indicate undervaluation (negative values), while yellow shades indicate overvaluation (positive values). Darker shades are applied for discrepancies of ±15% or greater, with 0% being the ideal outcome.

Condition C, which combined expert judgment with model assistance, yielded mixed results. Task 1 emerged as an outlier, as the model’s prediction deviated by more than $21\%$ MAPE. Most experts did not accurately assess this error, with predictions clustering around − 20%. This suggests that the deviation in the model’s initial estimate influenced the expert predictions. Task 1 exhibited the least variance in errors, suggesting cautious adjustments by experts or an unspoken consensus on the given price. The longer duration reflects a deliberate yet conservative approach—experts treated the first task seriously, aligned closely with the model’s suggestion, and hesitated to implement sizeable adjustments.

Task 2 revealed notable variability, with errors differing widely among participants. The largest deviations were from a few experts who overestimated the property value, while most clustered around $10\%$ to $15\%$ MAPE. Experts challenged the model’s predictions more in Task 2 than in Task 1, suggesting that they became more confident and critical following initial interactions with the model.

Task 3 showcased the potential of the hybrid approach, as six experts achieved errors under $5\%$ and none exceeded the $20\%$ threshold. This suggests significant accuracy gains when the model predictions align with expert intuition. Although the standalone performance of the model in Task 3 (9.2% error) was weaker than in Task 2 (2.5% error), the combination of expert judgment and model support in Task 3 nearly optimized the overall outcome.

In contrast, Task 4 produced the largest individual error by a participant in the entire experiment. Most experts performed well, with only two participants exceeding the MAPE threshold $20\%$. This suggests that most experts aligned closely with the accurate estimate of the model. However, the task also underlines the variability in expert predictions, as some participants notably deviated from both the model’s suggestion and the true value, underscoring the challenges in maintaining consistency and trust even when reliable model guidance is available.

Task 5 presented another outlier, with the model’s prediction deviating by approximately $31.5\%$ MAPE. Background analysis showed that the property had been sold at an unusually low price. Most experts agreed with the model, but two junior experts, Expert 4 and Expert 5, achieved very high accuracy by disagreeing. Possible reasons include familiarity with the property, a different approach, or luck.

Condition C illustrates both the potential and limitations of hybrid approaches. High-accuracy predictions ($< 5\%$ and $< 10\%$) increased in certain tasks, particularly Task 3, but outliers such as Tasks 1 and 5 underscored the persistent challenges of aligning expert judgment with model predictions. Experts are unlikely to challenge poor performance, but this must be seen in the context of the outliers’ placements. The first task may have provoked a weaker reaction, whereas the final task may have been influenced by mild fatigue—an effect not evident in Condition B.

Comparison of all conditions: In Table 7, the absolute average error of the model, each participant, and the overall participant average are compared across conditions. It is immediately apparent that the error decreases significantly when experts are allowed to use their state-of-the-art methods. However, this result is unsurprising, as experts in any field rely on their specialized tools to achieve high-quality results. Furthermore, when comparing Condition A with Condition B, it is evident that the model error remains quite stable, at approximately 10% MAPE. In Condition C, the model error increased by about 4%, and several participants showed a marked rise in average error.

Two outliers in Condition C, compared to one in Conditions A and B, can be attributed to random sampling bias and the nature of the underlying data distribution. Random sampling bias can result in an over-representation of extreme values due to statistical fluctuations inherent in the sampling process^47^. Furthermore, skewed or heavy-tailed distributions naturally produce more extreme values, increasing the likelihood of outliers^48^.

**Table 7:**
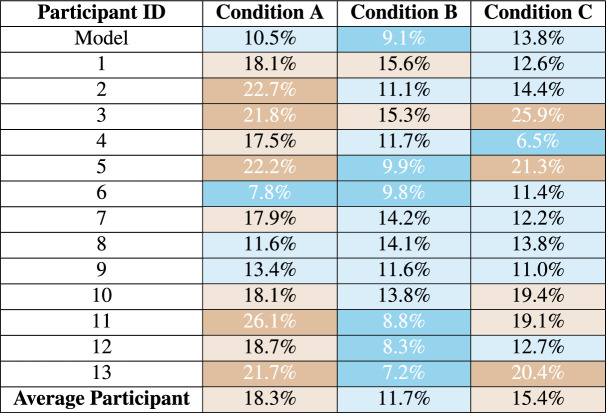
Comparison of prediction errors under different conditions. Dark cyan indicates lower errors (below 10%), light cyan for errors between 10-15%, light brown for errors between 15–20%, and dark brown for errors above 20%.

The adjusted MAPE framework enables more detailed analysis, highlighting very high accuracy and clearly delineating significant errors. For clarity, errors between 15–20% are not analyzed in depth; instead, the discussion focuses on high- and underperformance predictions.

**Table 8:** Performance of experts across different conditions and tasks, categorized by accuracy levels. Each task generated 13 results, corresponding to the 13 experts. The highest-performing results within the high-accuracy category (5%) and the 15% threshold category are highlighted. Additionally, tasks that resulted in the largest number of errors (>20%) are also marked for reference.

Condition	Task	$\le$ 5%	$\le$ 10%	$\le$ 15%	$\ge$ 20%
A	1	2	3	5	**8**
A	2	3	6	**8**	5
A	3	3	5	**8**	5
A	4	1	3	4	**9**
A	5	1	3	3	**10**
B	1	5	8	**9**	4
B	2	**6**	9	**10**	3
B	3	**0**	3	6	7
B	4	4	5	**9**	4
B	5	5	9	**11**	2
C	1	0	0	2	**11**
C	2	4	9	**10**	3
C	3	**6**	10	**13**	**0**
C	4	**6**	9	**9**	4
C	5	2	2	2	**11**

Table 8 illustrates the distribution of error categories between tasks, providing insight into the variability of performance among participants under different experimental conditions. The favorable categories are cumulative, which means that errors falling under 10% MAPE also include those under 5%, etc. As shown in the table, the majority of experts performed within the MAPE threshold of 15% in most tasks, underscoring the general robustness of their evaluations. However, key differences between conditions and tasks are evident:

By comparing **Condition A and Condition B**, the aforementioned increase in predictions below the 15% threshold becomes evident, accompanied by a corresponding decrease in large errors. Interestingly, Condition A is the only condition that consistently includes a high-accuracy prediction in every task. When comparing **Conditions A and C**, most experts demonstrated a clear improvement. This outcome is expected, as Condition C provided both a price suggestion and access to state-of-the-art tools. The combination of these resources appeared to enhance experts’ ability to refine their predictions, particularly for routine cases. However, the improvement observed in Condition B suggests that experts’ own methodologies and intuition had a stronger influence on their performance than the model’s assistance in Condition C. A direct comparison between **Conditions B and C** revealed different patterns in performance. The substantial impact of outliers in Condition C makes it difficult to draw meaningful conclusions. However, it can be stated that Condition C is the only condition that enabled experts to achieve accurate predictions without any large errors.

Removing outliers: Since the focus is on evaluating the model’s suitability as an assistance system, outliers must be addressed, and the conditions assessed under *comparable conditions*—that is, scenarios where model prediction errors in Conditions B and C are similar. To ensure comparability, the average model error across both conditions was equalized. For this, the two outlier objects from Condition C were removed. When only the single outlier point was removed from Condition B, the model’s average error remained higher than that of Condition C. Therefore, the second-worst prediction of the model in Condition B was also removed.

Table 9 provides a comparison of participant performance after removing outliers under Conditions B and C. The table reveals that 62% of participants experienced improved accuracy when transitioning from Condition B to Condition C. However, the average participant error increased slightly, from 9.1% in Condition B to 9.6% in Condition C. This suggests that while many participants benefited from model assistance, the hybrid approach introduced challenges for some participants, leading to a significant increase in error for certain individuals.

**Table 9:** Performance metrics after outlier removal for Conditions B and C This table compares participant accuracy under Conditions B (expert-only methods) and C (hybrid collaboration) after removing outliers.

ID	AVG B	AVG C	ACC improved
Model	5.5%	5.4%	
1	7.8%	4.6%	Yes
2	11.2%	3.6%	Yes
3	11.3%	21.9%	No
4	15.3%	2.9%	Yes
5	3.2%	29.4%	No
6	3.8%	4.8%	No
7	17.4%	4.0%	Yes
8	17.7%	4.2%	Yes
9	5.1%	4.0%	Yes
10	4.4%	12.5%	No
11	9.8%	9.6%	Yes
12	10.5%	8.2%	Yes
13	1.5%	15.8%	No
Average part	9.1%	9.6%	61.5%

**Fig. 3:**
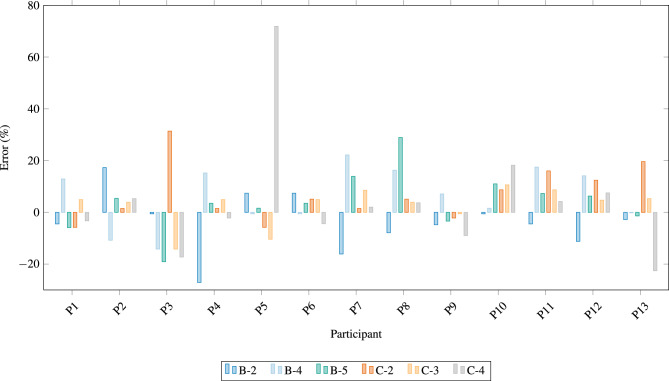
This figure compares the performance of participants across outlier-cleaned tasks between Condition B and Condition C, highlighting the differences in task completion rates after removing extreme values. Each bar represents a participant’s error across different conditions, with positive values indicating overestimation and negative values underestimation.

Figure 3 shows the remaining prediction errors after outliers were removed to mitigate the bias introduced by the poor performance of the model on certain tasks. Five experts experienced decreased performance from Condition B to Condition C, four of them showing substantial declines. Among these, **Expert 13**, who excelled in Condition B with errors less than $5\%$ for all tasks, showed a significant decrease in accuracy in Condition C. This suggests that the model predictions contradicted their judgment, leading to less accurate estimates. Highly experienced experts may rely more on their knowledge and intuition, which, while valuable, can occasionally conflict with accurate model predictions. This behavior could arise from confidence in their expertise, a deeper understanding of contextual nuances, or skepticism toward automated assistance. Although this reliance can be beneficial, it may result in suboptimal outcomes when the model’s guidance is accurate but challenges the expert’s intuition.

For the three other experts whose performance decreased significantly in Condition C, the number of large errors increased by one, and their overall prediction quality worsened. This was particularly evident in **Experts 3, 5**, and **10**. These cases suggest that the model’s input may have disrupted decision-making processes, leading to conflicts rather than improvements. This is reflected in Experts 3 and 10 rating the proposed price information as moderately helpful, while Expert 5 found it not helpful at all. Expert 5 recorded an error of nearly 29% in Condition C compared to 3.2% in Condition B, attributed to a substantial discrepancy in one specific task.

Conversely, participants who improved in Condition C exhibited reduced error variability, indicating that model assistance helped streamline their predictions. In particular, Participant 7 achieved a marked improvement, with errors decreasing from 17.4% in Condition B to 4.0% in Condition C, demonstrating the potential of the model to enhance decision-making when experts effectively integrate its output.

**Fig. 4:**
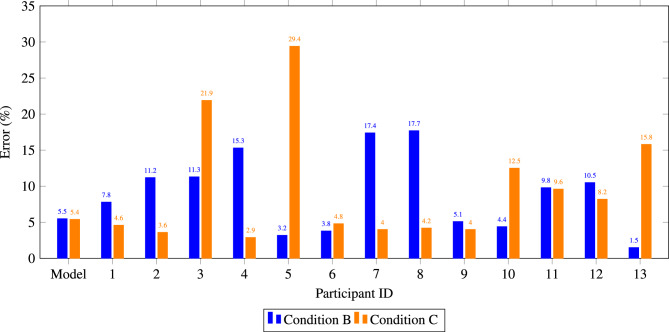
Performance metrics for outlier removed Condition B and C. Each bar represents the MAPE for individual participants over the Condition. The chart showcase participants whose performance improved with model assistance (Condition C) and those who experienced increased errors, emphasizing the variability in hybrid approach effectiveness.

Figures in Fig. 4 emphasize the nuanced dynamics of human–machine collaboration. Although the hybrid approach generally aids accuracy, it may occasionally conflict with the intuition of highly experienced experts, leading to counterproductive adjustments.

The five participants whose errors increased when using the assisted system showed notable discrepancies in Condition C. Upon examining their individual errors, it becomes evident that three of them exceeded the suggested error thresholds for a given price, as shown in Table 10. In the table, individual errors for each task under Condition C can be observed, along with their average absolute errors for Conditions B and C, excluding the outlier data points.

Four major outliers in expert predictions were identified across conditions, complicating the qualitative interpretation but providing important information. The largest outlier was from Expert 5, likely due to an overreliance on internal tools and a misjudgment during the task. As a junior expert, this individual may have overestimated their methodology. This aligns with the fact that Expert 5 argued against the given price in two of the three remaining tasks, with the most considerable error occurring in the fourth task, where their prediction resulted in an error of nearly 72%. This significant discrepancy alone caused the overall error of Expert 5 in Condition C to rise to almost 22%. Notably, however, this Expert performed very well in Condition B, suggesting that the current assistance system may not be well-suited to their needs. The second largest error came from Expert 3, who reported high mental demand in the NASA TLX self-assessment. Despite their 22 years of experience, their performance may have been affected by psychological stress or the discomfort of the experimental setup. Similarly, Expert 3 argued against the given price in all three remaining tasks, and their error in the second task of Condition C exceeded 34%, contributing to an overall error of almost 16.4% in this condition. Expert 13 also produced two outliers in Condition C, probably due to a rushed approach during the latter part of the experiment. This suggests that fatigue or decreased attention to detail can notably affect performance in prolonged tasks. Correspondingly, Expert 13 argued against the given price in two of the three remaining tasks. While their errors were not as extreme as those of Experts 3 and 5, they were still notable. However, like Expert 5, Expert 13 performed well in Condition B, further supporting the idea that the assistance system may not align well with their decision-making processes.

**Table 10:** Participant performance condition C of tasks 2, 3, and 4; average absolute error of condition B and C with outlier observations removed. Mayor deviation from expert from the models predictions are highlighted.

Participant	Task 2	Task 3	Task 4	AVG B	AVG C
3	**34.1%**	− **14.2%**	− **17.3%**	11.3%	21.9%
5	− 5.8%	− **10.4%**	**71.9%**	3.2%	29.4%
6	5.1%	4.9%	− 4.4%	3.8%	4.8%
10	8.7%	10.6%	18.2%	4.4%	12.5%
13	**19.6%**	5.3%	− **22.6%**	1.5%	15.8%

These findings bring attention to the complexities of integrating model assistance into expert workflows. Although automation can provide valuable support, its effectiveness depends on its alignment with expert intuition and workflows. The cases of Experts 3, 5, and 13 illustrate that automated guidance can sometimes create conflicts that degrade performance rather than enhance it.

This variability underscores the importance of individual factors such as experience, confidence, and familiarity with data-driven tools. While a slight tendency suggests that junior participants may benefit more from model assistance, no statistically significant seniority effects were identified. Understanding how expertise level interacts with hybrid support systems therefore remains a critical direction for future research.

### Time

**Fig. 5:**
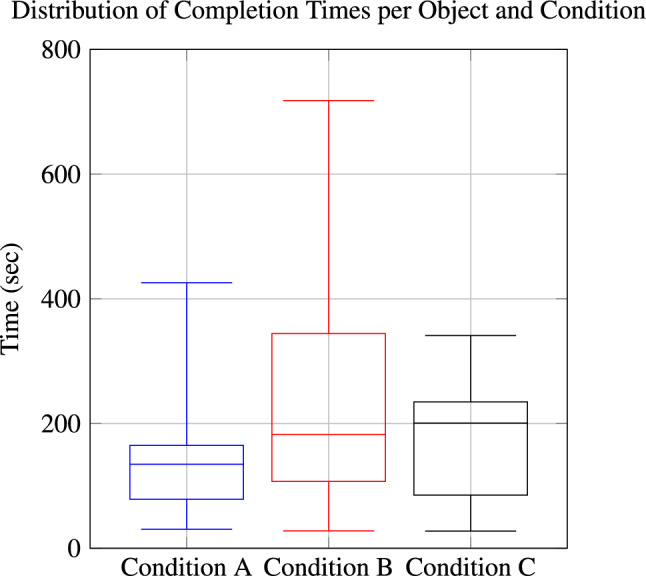
Boxplot of average completion times per object across conditions A, B, and C.

Comparing task completion times between conditions reveals two key observations. Condition A was, with a few exceptions, completed fastest among experts (see Fig. 5). This may be due to the limited information available, which requires less data processing. In contrast, for other conditions, longer times are due to a more comprehensive data analysis.

Secondly, the total time varied significantly between the participants. The fastest expert completed each task in under a minute, with a total duration of less than 15 min. The slowest expert took approximately one and a half hours in total, with individual tasks lasting up to 12 min.

In addition to variability in accuracy, task completion times highlight differences in individual working styles and the challenges of integrating automated tools with expert workflows. Faster experts relied on intuition and minimal data analysis, whereas slower participants employed more detailed methods. However, even the fastest expert was vastly outperformed by the model, which generated predictions in a fraction of a second. The trade-off between accuracy and efficiency highlights the need for strategies that balance both.

### Information evaluation

The sole common element across all three conditions is the object information, which serves as the consistent basis for comparison. Table 11 shows the relative importance of different types of information under each condition. The consistent valuation of object information indicates that, while additional data can enhance accuracy, certain fundamental details remain crucial in all scenarios. The data in Table 11 show that object information is valued most by experts under conditions with limited contextual data. Sociodemographic information was deemed insignificant in Condition A, where infrastructure data and object information were considered the most relevant.

A broader range of information can be compared for Conditions B and C, as both include similar data apart from sqm price prediction in Condition C. Despite this, the object information is valued lower than in Condition A. Comparable prices and the timeline for square meter prices are valued higher in Condition C than in Condition B. However, the value placed on experts’ own online research was lower in Condition C than in Condition B, possibly indicating a higher reliance on the model’s assistance in Condition C.

**Table 11:** Perceived relevance of different categories of information under different experimental conditions (1 = Good, 5 = Poor, 3 = Average).

Type	Cond A	Cond B	Cond C
Object information	2.15	2.23	2.23
Neighborhood information	2.62		
Socio-demographic	3.62		
Educational level	3.62		
Infrastructure information	2.23		
Comparison transactions		2.31	2.15
$\hbox {m}^2$ Price time series		2.31	2.15
Own research		2.31	2.46
Estimated $\hbox {m}^2$ Price			2.62

### Workload

The NASA TLX workload self-evaluation revealed no significant differences. Across the three conditions, the six assessed parameters remained stable. Only minimal variations were observed across conditions.

**Table 12:** NASA TLX assessment average results across conditions (1 = very low, 5 = very high).

Condition	A	B	C	Overall
Mental demands	3.38	3.38	3.31	3.36
Physical demands	1.54	1.92	1.77	1.74
Temporal demands	2.85	2.92	2.69	2.82
Performance	3.31	3.38	3.38	3.36
Effort	2.38	2.69	2.92	2.67
Frustration	2.54	2.62	2.54	2.56

Table 12 shows that NASA TLX scores remained consistent across all conditions. Despite differences in task completion times and working styles, no notable variations in perceived demand were observed. This indicates that the task design was balanced. In particular, mental demand and performance emerged as key factors. Although tools and information can improve prediction accuracy, they do not impose a greater burden on experts. This finding highlights the importance of designing assistance systems that align with expert workflows.

### Summary of results and research questions

The study was guided by four research questions. First, a realistic experimental setup was created by embedding the tasks in the actual work environment of professional appraisers, ensuring high ecological validity while maintaining experimental control. Second, when both the model and experts were provided with the same structured information, the XGBoost model achieved an accuracy comparable to, or even exceeding, human performance, confirming its strong predictive power under standardized conditions. Third, when applying their usual professional assessment routines, the experts’ performance varied more widely, suggesting that individual judgment and contextual thinking remain crucial factors. Finally, collaboration between experts and the model yielded mixed results: while some participants benefited from the model’s support, others were adversely affected, suggesting that the effectiveness of automated assistance depends strongly on user experience, trust, and workflow compatibility.

**Fig. 6:**
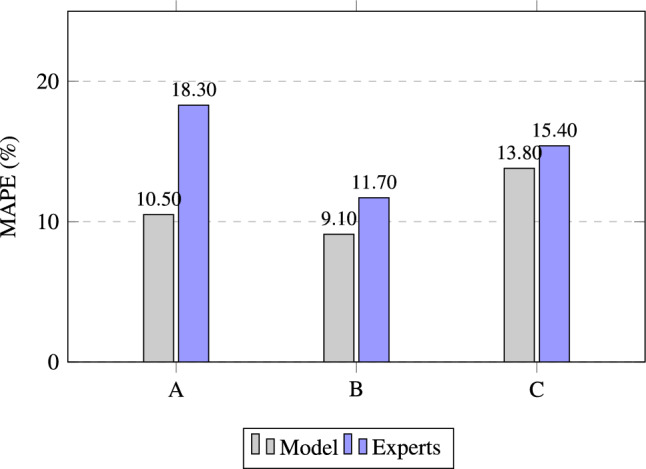
Comparative accuracy (MAPE %) of experts and the model across Conditions A–C. The model remains below the 15% threshold in all conditions–though highest in C due to outliers—and outperforms experts overall. Expert accuracy improves from A to B and is closest to the model in the hybrid Condition C.

Figure 6 illustrates the comparative performance of experts and the model across the experimental conditions. While expert performance improved markedly when using their standard tools (Condition B), the hybrid setup (Condition C) showed the smallest performance gap between human and machine predictions, indicating potential for effective collaboration when support is appropriately integrated.

**Fig. 7:**
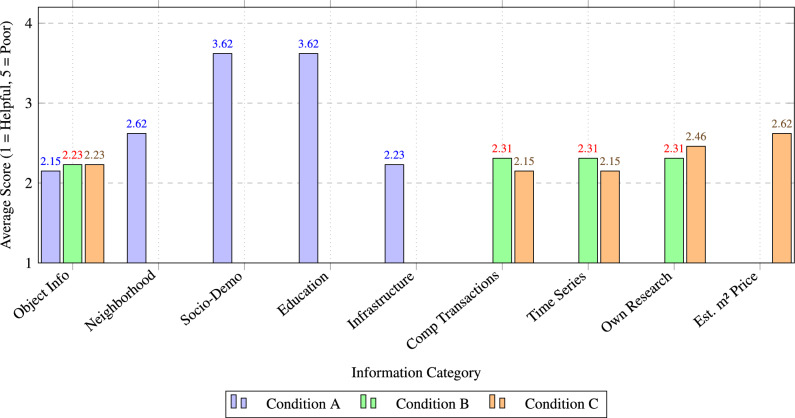
Perceived relevance of different categories of information under different experimental conditions (lower = more helpful). The diagram compares how experts rated the usefulness of different types of information when performing evaluation tasks under conditions A–C. In all constellations, object information remained consistently important, underscoring its fundamental role in evaluation decisions. In contrast, socio-demographic and educational data were considered least relevant under Condition A. The inclusion of the model’s suggested price (average value = 2.62) shows moderate perceived usefulness, suggesting that price suggestions are perceived as helpful.

Figure 7 summarizes the perceived relevance of different information categories. Condition A captures general human information prioritization, while Conditions B and C emphasize how experts use data when supported by analytical tools or model predictions. These results indicate that while the model introduces new decision cues, fundamental object and price information—along with experts’ own research–remain the most trusted sources for professional judgment.

The results suggest that ML-based prediction models can serve as helpful decision-making aids for real estate professionals in practice—in particular, when it comes to quickly estimating prices or determining values in unfamiliar markets. Model outputs should be regarded as well-founded guides rather than objective truths, as human expertise remains indispensable for context-specific assessments.

Cases in which collaboration failed highlight the potential influence of cognitive biases and the consequences of excessive dependence. As no qualitative feedback was collected, these aspects remain open for future investigation: Future research should explore how adaptive support and improved model transparency can mitigate these effects and enable more effective collaboration between humans and ML.

## Conclusion and future work

The proposed model matched or exceeded the performance of field experts in predicting real estate prices. This finding validates the model’s potential as a valuable tool in real-world scenarios. However, challenges persist in accurately addressing outlier properties. Addressing these challenges is critical to enhancing the model’s practical utility.

The findings highlight the value of hybrid approaches, in which machine learning models supplement expert predictions, particularly in scenarios involving incomplete or complex data. While the model consistently performed well, expert intuition and local knowledge remain essential. Future development should focus on integrating model assistance effectively without undermining expert confidence. Although model assistance did not uniformly improve expert assessments, it shows promise as an efficient tool for generating price anchor points in areas less familiar to experts.

Another key finding is that sociodemographic information appears less relevant to price evaluation for experts than it does for the model. Experts tend to prioritize other characteristics, guided by their experience and intuition. In particular, two participants who relied heavily on intuition achieved high accuracy while completing tasks rapidly. This raises questions about balancing data-driven decision-making with intuitive judgment, emphasizing the need for careful calibration of model outputs to effectively supplement human expertise. Future studies should also include confidence calibration measures to assess how accurately experts estimate their own certainty relative to model performance. Understanding this alignment between human confidence and model accuracy could provide valuable insights into when and how hybrid decision-making is most effective.

The experiments confirm that machine predictions can match expert performance when sufficient data are available. However, as an assisting tool, the model’s impact on expert performance was mixed, particularly when dealing with outliers. Such observations underscore the need for further refinement in handling atypical data and more comprehensive testing of the model’s role as a decision-support tool. Transparency in future studies, such as informing experts that model assistance is being tested, could improve trust and provide clearer insights into how the tool influences decisions. Future work may also incorporate visual information from the urban environment to better replicate the information available to experts during the assessment. The inclusion of such elements could improve prediction accuracy and more closely mirror the human approach while maintaining the spatial focus of the model.

Increasing the number of participants and conducting multiple studies would enable a meta-analysis to identify broader trends, refine predictive models, and reveal insights not visible in individual studies. This approach would provide a deeper understanding of the interactions between expert judgments and machine learning in real estate price prediction and could help mitigate the influence of outliers.

A key limitation of the current study is the small number of evaluation points per test condition, which results from the study’s focus on ecological validity. This was compounded by the limited availability of highly qualified experts and the time-consuming nature of each assessment, which sometimes took about an hour per condition 5. While this design ensured realistic working conditions and high-quality input, it naturally constrained sample size and limited generalizability. For this reason, no classical inference methods were used (see section Participants above). Future research should, therefore, expand the scope of the study, particularly by aiming to testing of a larger group of experts, performing similar work in other urban environments and including larger and more diverse datasets to improve statistical robustness. Urban environments, with their higher transaction density and more uniform market dynamics, are particularly well suited for developing robust models. Rural areas may seem promising, but their sparse data points and variability in property characteristics make it difficult to develop reliable models.

Future studies should also investigate the types and quantities of information provided to experts to optimize prediction accuracy. Providing clear and interpretable model results could help build trust and improve understanding among experts. In addition, incorporating local knowledge and contextual data could improve predictions, especially for experts who may not be familiar with specific areas. Small-scale spatial knowledge may be particularly beneficial in such cases.

A promising direction for future work is exploring adaptive assistance systems that tailor their support to the characteristics of the property and the previous performance of the expert. Incorporating uncertainty quantification methods, such as confidence intervals or probabilistic guidance, could help standardize decision-making. Furthermore, machine learning models that learn from expert variability could identify patterns in discrepancies, offering targeted interventions to harmonize predictions. By reducing variability, such systems could lead to improved decision-making and greater confidence in predictive models in diverse applications.

## Supplementary Information

Supplementary Information 1.

Supplementary Information 2.

## Data Availability

Data and materials supporting the findings of this study are made available through the university’s data repository (DOI: https://doi.org/10.48436/zs6cy-6t304). This includes the complete questionnaire used for the experiments, all computed geospatial features, the full set of questionnaire responses, model results and real labels, and the machine learning model scripts. Transaction-level features and trained machine learning models cannot be publicly shared due to commercial restrictions, as they are proprietary to an external company.

## References

[1] Nijskens, R., Lohuis, M., Hilbers, P. & Heeringa, W. *Hot Property: The Housing Market in Major Cities* (Springer Nature, 2019).

[2] Harré, M. S. & Zaitouny, A. Detecting criticality in complex univariate time-series: A case study of the us housing market crisis and other markets. *Expert Syst. Appl.* **211**, 118437 (2023).

[3] Meek, R. L. The interpretation of the’’ tableau economique’’. *Economica* **27**, 322–347 (1960).

[4] Lisec, A. & Navratil, G. The austrian land cadastre: From the earliest beginnings to the modern land information system. *Geodetski vestnik*. **58** (2014).

[5] Rosen, S. Hedonic prices and implicit markets: Product differentiation in pure competition. *J. Polit. Econ.* **82**, 34–55. 10.1086/260169 (1974).

[6] Helbich, M., Jochem, A., Mücke, W. & Höfle, B. Boosting the predictive accuracy of urban hedonic house price models through airborne laser scanning. *Comput. Environ. Urban Syst.* **39**, 81–92. 10.1016/j.compenvurbsys.2013.01.001 (2013).

[7] Abidoye, R. B. & Chan, A. P. Improving property valuation accuracy: A comparison of hedonic pricing model and artificial neural network. *Pac. Rim Prop. Res. J.* **24**, 71–83. 10.1080/14445921.2018.1436306 (2018).

[8] Rey-Blanco, D., Zofío, J. L. & González-Arias, J. Improving hedonic housing price models by integrating optimal accessibility indices into regression and random forest analyses. *Expert Syst. Appl*. **235**, 10.1016/j.eswa.2023.121059 (2024).

[9] Sellam, Z. A., Distante, C., Taleb-Ahmed, A. & Mazzeo, P. L. Boosting house price estimations with multi-head gated attention. *Expert Syst. Appl.* **259**, 125276. 10.1016/j.eswa.2024.125276 (2025).

[10] Baur, K., Rosenfelder, M. & Lutz, B. Automated real estate valuation with machine learning models using property descriptions. *Expert Syst. Appl.* **213**, 119147 (2023).

[11] Kmen, C., Navratil, G. & Giannopoulos, I. Location, location, location: The power of neighborhoods for apartment price predictions based on transaction data. *ISPRS Int. J. Geo Inf.* **13**, 425 (2024).

[12] Wang, D., Yang, Q., Abdul, A. & Lim, B. Y. Designing theory-driven user-centric explainable AI. *Conf. Hum. Factors Comput. Syst. Proc.*10.1145/3290605.3300831 (2019) (**(Association for Computing Machinery**).

[13] Alberdi, E., Strigini, L., Povyakalo, A. A. & Ayton, P. Why are people’s decisions sometimes worse with computer support? In *Lecture Notes in Computer Science (including subseries Lecture Notes in Artificial Intelligence and Lecture Notes in Bioinformatics)*, vol. 5775 LNCS, 18–31. 10.1007/978-3-642-04468-7_3 (2009).

[14] Kaur, H. *et al.* Interpreting Interpretability: Understanding Data Scientists’ Use of Interpretability Tools for Machine Learning. In *Conference on Human Factors in Computing Systems—Proceedings*, 1–14. 10.1145/3313831.3376219 (Association for Computing Machinery, 2020).

[15] Bayer, S., Gimpel, H. & Markgraf, M. The role of domain expertise in trusting and following explainable ai decision support systems. *J. Decis. Syst.* **32**, 110–138 (2022).

[16] Schmidt, P., Biessmann, F. & Teubner, T. Transparency and trust in artificial intelligence systems. *J. Decis. Syst.* **29**, 260–278 (2020).

[17] Cecil, J., Lermer, E., Hudecek, M. F., Sauer, J. & Gaube, S. Explainability does not mitigate the negative impact of incorrect ai advice in a personnel selection task. *Sci. Rep.* **14**, 9736 (2024). 38679619 10.1038/s41598-024-60220-5 PMC11056364

[18] Cummings, M. M. Man versus machine or man+ machine?. *IEEE Intell. Syst.* **29**, 62–69 (2014).

[19] Bansal, G., Wu, T. & Zhou, J. Does the whole exceed its parts? The effect of ai explanations on complementary team performance. In *Conference on Human Factors in Computing Systems—Proceedings*, 1–16. 10.1145/3411764.3445717 (Association for Computing Machinery, 2021).

[20] de Visser, E. J., Pak, R. & Shaw, T. H. From ‘automation’ to ‘autonomy’: the importance of trust repair in human–machine interaction. *Ergonomics* **61**, 1409–1427. 10.1080/00140139.2018.1457725 (2018). 29578376 10.1080/00140139.2018.1457725

[21] Dellermann, D. *et al.* The future of human-ai collaboration: a taxonomy of design knowledge for hybrid intelligence systems. arXiv preprint arXiv:2105.03354 (2021).

[22] Green, B. & Chen, Y. Disparate interactions: An algorithm-in-the-loop analysis of fairness in risk assessments. In *FAT* 2019—Proceedings of the 2019 Conference on Fairness, Accountability, and Transparency*, 90–99. 10.1145/3287560.3287563 (Association for Computing Machinery, Inc, 2019).

[23] Miller, T. *Explanation in artificial intelligence: Insights from the social sciences*. 10.1016/j.artint.2018.07.007 (2019).

[24] Northcraft, G. B. & Neale, M. A. Experts, amateurs, and real estate: An anchoring-and-adjustment perspective on property pricing decisions. *Organ. Behav. Hum. Decis. Process.* **39**, 84–97. 10.1016/0749-5978(87)90046-X (1987).

[25] Ni, F., Arnott, D. & Gao, S. The anchoring effect in business intelligence supported decision-making. *J. Decis. Syst.* **28**, 67–81 (2019).

[26] Birkeland, K. B., D’Silva, A. D., Füss, R. & Oust, A. The predictability of house prices:’’ human against machine’’. *Int. Real Estate Rev.* **24**, 139–183 (2021).

[27] Poursabzi-Sangdeh, F., Goldstein, D. G. & Hofman, J. M. Manipulating and measuring model interpretability. In *Conference on Human Factors in Computing Systems—Proceedings*, 1–52. 10.1145/3411764.3445315 (Association for Computing Machinery, 2021).

[28] McGrath, S., Mehta, P., Zytek, A., Lage, I. & Lakkaraju, H. When Does Uncertainty Matter?: Understanding the Impact of Predictive Uncertainty in ML Assisted Decision Making. arXiv preprint arXiv:2011.06167 (2020).

[29] Agarwal, N., Moehring, A., Rajpurkar, P. & Salz, T. Combining Human Expertise with Artificial Intelligence: Experimental Evidence from Radiology. Tech. Rep., National Bureau of Economic Research, Cambridge, MA (2023). 10.3386/w31422.

[30] Lai, V., Chen, C., Smith-Renner, A., Liao, Q. V. & Tan, C. Towards a science of human–AI decision making: An overview of design space in empirical human-subject studies, 1369–1385 (2023).

[31] Chen, T. & Guestrin, C. XGBoost: A scalable tree boosting system. In *Proceedings of the ACM SIGKDD International Conference on Knowledge Discovery and Data Mining*, vol. 13-17-August-2016, 785–794. 10.1145/2939672.2939785 (Association for Computing Machinery, 2016).

[32] Geerts, M., Vanden Broucke, S. & De Weerdt, J. A survey of methods and input data types for house price prediction. *ISPRS Int. J. Geo Inf.* **12**, 200 (2023).

[33] Li, S., Jiang, Y., Ke, S., Nie, K. & Wu, C. Understanding the effects of influential factors on housing prices by combining extreme gradient boosting and a hedonic price model (xgboost-hpm). *Land* **10**, 533 (2021).

[34] De Nadai, M. & Lepri, B. The economic value of neighborhoods: Predicting real estate prices from the urban environment. In *2018 IEEE 5th International Conference on Data Science and Advanced Analytics (DSAA)*, 323–330 (IEEE, 2018).

[35] Jha, S. B., Babiceanu, R. F., Pandey, V. & Jha, R. K. Housing market prediction problem using different machine learning algorithms: A case study. *arXiv preprint* arXiv:2006.10092 (2020).

[36] Avanijaa, J. et al. Prediction of house price using xgboost regression algorithm. *Turk. J. Comput. Math. Educ. (TURCOMAT)* **12**, 2151–2155 (2021).

[37] Forbes, D. Blinding: An essential component in decreasing risk of bias in experimental designs. *Evid.-Based Nurs.* **16**, 10.1136/eb-2013-101382 (2013). 10.1136/eb-2013-101382 23696228

[38] Wood, L. et al. Empirical evidence of bias in treatment effect estimates in controlled trials with different interventions and outcomes: meta-epidemiological study. *BMJ* **336**, 601–605 (2008). 18316340 10.1136/bmj.39465.451748.AD PMC2267990

[39] Hróbjartsson, A., Emanuelsson, F., Skou Thomsen, A. S., Hilden, J. & Brorson, S. Bias due to lack of patient blinding in clinical trials. a systematic review of trials randomizing patients to blind and nonblind sub-studies. *Int. J. Epidemiol.* **43**, 1272–1283 (2014). 24881045 10.1093/ije/dyu115 PMC4258786

[40] Probst, P. et al. Blinding in randomized controlled trials in general and abdominal surgery: protocol for a systematic review and empirical study. *Syst. Rev.* **5**, 1–6 (2016). 27012940 10.1186/s13643-016-0226-4 PMC4806514

[41] Vaccaro, M., Almaatouq, A. & Malone, T. When combinations of humans and ai are useful: A systematic review and meta-analysis. *Nat. Hum. Behav.* **8**, 2293–2303 (2024). 39468277 10.1038/s41562-024-02024-1 PMC11659167

[42] Makin, T. R. & Orban de Xivry, J.-J. Ten common statistical mistakes to watch out for when writing or reviewing a manuscript. *Elife* **8**, e48175 (2019). 31596231 10.7554/eLife.48175 PMC6785265

[43] Hopkin, C. R., Hoyle, R. H. & Gottfredson, N. C. Maximizing the yield of small samples in prevention research: A review of general strategies and best practices. *Prev. Sci.* **16**, 950–955 (2015). 25578307 10.1007/s11121-014-0542-7 PMC4500750

[44] D’Arrigo, G. et al. Common mistakes in biostatistics. *Clin. Kidney J.* **17**, sfae197 (2024). 39165900 10.1093/ckj/sfae197 PMC11333961

[45] Hancock, P. A. & Meshkati, N. *Human Mental Workload*, 52 (North-Holland Amsterdam, 1988).

[46] Lewis, C. *Industrial and Business Forecasting Methods: A Practical Guide to Exponential Smoothing and Curve Fitting* (Butterworth scientific (Butterworth Scientific, 1982).

[47] Tripepi, G., Jager, K. J., Dekker, F. W. & Zoccali, C. Selection bias and information bias in clinical research. *Nephron Clin. Pract.* **115**, c94–c99 (2010). 20407272 10.1159/000312871

[48] Dallah, D., Sulieman, H. & Alzaatreh, A. Relative range for skewed distributions: a tool for outlier detection. *Gulf J. Math.* **16**, 298–312 (2024).

