# Supplementary material for: Evaluating human–machine collaboration through a comparative analysis of experts, machine learning, and hybrid approaches in real estate valuation
Source: Sci Rep. 2026 Jan 17;16:4044. doi: 10.1038/s41598-025-34099-9 (PMC12855191; doi:10.1038/s41598-025-34099-9)
Supplement: Supplementary file 1 — Supplementary Information 1. [file 41598_2025_34099_MOESM1_ESM.pdf]

RESPONSIBLE
RESEARCH PRACTICES

TUW Research Ethics Committee

Research Ethics Review

15/03/2024

Title of Project: Evaluating Performance in Real Estate Prediction

Name of Researcher: Christopher Kmen

Number: 048_022024_TUWREC

Reviewed in remote in February and March 2024 by 3 members of the Research Ethics Committee.

In general:

- The reviewers considered the research well planned overall. Some questions were raised.
- Participants are from the researcher's workplace and employed by the company funding the research. This relates to individual and personal information but also to the results of the study and its use by the funding company. Is there a possibility that the data/results will be used to assess performance of the team/unit?
- There is a slight concern regarding privacy (the PI has access to all the data). The question is whether personal data is really necessary for the experiment or whether it can be reduced. The researcher describes the potential apprehension of participants that they could feel evaluated, and this is mitigated by making the data available only to the research team. But since the PI works for the company as well, this could be a problem later on. Just imagine, the PI after PhD gets a management position in the company and remembers the bad performance of some participants from the company in the experiment.
- It appears that there is an element of deception as the participants will not be told that they are competing against AI. Debriefing has not been mentioned but would need to be taken into account. The question was raised what the motivation for not revealing the research setting would be.
- Moreover, there was a question raised about potential pressure to participate as the research takes place in a work place.
- It is not clear where the survey takes place; online or at the premises of TU, for example?
- No consent form has been attached.

Recommendations:

With regard to the participants from the researcher's workplace, any questions about the use of data and results need to be addressed early on via a detailed information in the informed consent documentation. A q&a session could also be arranged. Options should b explored to find a way to recruit experts from outside the funding company.

The researcher is reminded that an informed consent procedure is needed when participants are engaged in the research. The researcher is asked to refer to the TU Wien Guidance

RESPONSIBLE
RESEARCH PRACTICES

Document on Informed Consent in drafting the informed consent documentation.[1] Participants should be informed that they can withdraw their consent at any time and interrupt their participation without any consequences.

The researcher is asked to consider any dependencies between the researchers/supervisors and study participants (students and expert colleagues). Any feeling of coercion in participants should be avoided.

The researcher is recommended to include inclusion/exclusion criteria to the study design; who can participate, who should not? This is to be communicated also in the recruitment process.

Only those data that are necessary for the research should be collected. The duration of keeping the data should be defined (for example, 5 after the completion of the thesis). This should not be an indefinite time.

Information on research ethics review

The TU Wien Research Ethics Committee gives recommendations to researchers who seek research ethics guidance on their proposed research that involves human participants. The submission to ethics peer review is voluntary, the responsibility to implement the given guidance rests on the researcher.

On behalf of the reviewers

Dr. Marjo Rauhala

Chair of the TU Wien REC, Head of Unit of Responsible Research Practices

[1] TU Wien Guidance Document, Informed Consent: Good Practice Recommendations. https://www.tuwien.at/index.php?eID=dms&s=4&path=Documents/GDPR%20Guidelines%20and%20FAQs/Research_Projects_Guidance_Document_Informed_Consent.pdf

Statement to TUW Research Ethics Committee Review

Title of Project: Evaluating Performance in Real Estate Prediction

Name of Researcher: Christopher Kmen

Number: 048_022024_TUWREC

General

- *Participants are from the researcher's workplace and employed by the company funding the research. This relates to individual and personal information but also to the results of the study and its use by the funding company. Is there a possibility that the data/results will be used to assess performance of the team/unit?*

- *There is a slight concern regarding privacy (the PI has access to all the data). The question is whether personal data is really necessary for the experiment or whether it can be reduced. The researcher describes the potential apprehension of participants that they could feel evaluated, and this is mitigated by making the data available only to the research team. But since the PI works for the company as well, this could be a problem later on. Just imagine, the PI after PhD gets a management position in the company and remembers the bad performance of some participants from the company in the experiment.*

I will tackle both points simultaneously, as they are closely intertwined. Within my company, my department operates independently from the divisions that include the experts in question. Also the likelihood of ascending to a management role is slim, given the company's flat organizational structure. Moreover, assuming a management position in the expert's division would necessitate a fundamental shift in my career path. Such roles are typically reserved for individuals with extensive experience as real estate agents or appraisers, which diverges from my background.

- *It appears that there is an element of deception as the participants will not be told that they are competing against AI. Debriefing has not been mentioned but would need to be taken into account. The question was raised what the motivation for not revealing the research setting would be.*

During our discussion on experiment planning, a potential bias related to Machine Learning emerged, particularly from individuals not thoroughly acquainted with this technology. It has been observed in other studies that participants' performance may be negatively affected by their awareness of competing against AI. To mitigate this bias, we will not disclose the AI component of the study during the experiments. However, we will include a debriefing at the conclusion of the experiment to clarify and address any questions or concerns.

- *Moreover, there was a question raised about potential pressure to participate as the research takes place in a work place.*

The experiments will also be conducted on a voluntary basis in the workplace. I will reach out to the team leaders of both expert groups, requesting their teams' participation and emphasizing the voluntary nature of the study. This approach has received approval from one of the CEOs, and also HR is informed about this initiative. Furthermore, I will post an announcement on the internal communication platform to invite anyone interested in participating.

- *It is not clear where the survey takes place; online or at the premises of TU, for example?*

The study is designed to be conducted online. However, if requested by the participants, I am willing to accompany the process and provide assistance as needed.

- *No consent form has been attached.*

The informed consent document has been revised and submitted to the Research Ethics Committee (REC) for review.

Addressing Recommendations

- *With regard to the participants from the researcher's workplace, any questions about the use of data and results need to be addressed early on via a detailed information in the informed consent documentation. A q&a session could also be arranged. Options should be explored to find a way to recruit experts from outside the funding company.*
- *The researcher is reminded that an informed consent procedure is needed when participants are engaged in the research. The researcher is asked to refer to the TU Wien Guidance Document on Informed Consent in drafting the informed consent documentation. 1 Participants should be informed that they can withdraw their consent at any time and interrupt their participation without any consequences.*

As previously mentioned, a revised version of the informed consent document has been sent to the board. This document was updated in accordance with the TU Wien Guidance Document to ensure compliance and alignment with established standards. I will offer the option of a Q&A session if there is a need for it.

- *The researcher is asked to consider any dependencies between the researchers/supervisors and study participants (students and expert colleagues). Any feeling of coercion in participants should be avoided.*

We will take this into consideration. However, due to the organizational structure of the company, dependencies between researchers or supervisors and participants are unlikely. Additionally, for non-expert participants, there are minimal concerns regarding coercion. The questionnaire is distributed via email as an anonymous link and ensures anonymity for those participating, further mitigating any potential issues of undue influence.

- *The researcher is recommended to include inclusion/exclusion criteria to the study design; who can participate, who should not? This is to be communicated also in the recruitment process.*

The criteria for participation are established by the distribution mechanisms. Apart from an age limitation included within the questionnaire, everyone is invited to participate.

- *Only those data that are necessary for the research should be collected. The duration of keeping the data should be defined (for example, 5 after the completion of the thesis). This should not be an indefinite time.*

This issue is covered in the informed consent form. Raw data will be stored only for the duration necessary.
