# Supplementary material for: Evaluating human–machine collaboration through a comparative analysis of experts, machine learning, and hybrid approaches in real estate valuation
Source: Sci Rep. 2026 Jan 17;16:4044. doi: 10.1038/s41598-025-34099-9 (PMC12855191; doi:10.1038/s41598-025-34099-9)
Supplement: Supplementary file 2 — Supplementary Information 2. [file 41598_2025_34099_MOESM2_ESM.pdf]

Supplementary Material

December 13, 2025

Appendix A: Feature List

To provide an overview of all all features used in the modeling process, Table S1 summarizes the complete set of characteristics that was applied in the final data set. The table groups features by thematic categories, indicates their dimensionality, and describes how each class is linked to the target observations.

Feature	Count	Description	Form of integration
Transaction	6	Usable area, investor, penthouse, newly built and transaction date, subsidized	Base data points; individual
Seller/Buyer Class	6	based on Transaction classification	individual; one-hot-encoding
Size Class	4	based on Transaction classification	individual; one-hot-encoding
Transaction count per object	1	No. Transaction per Object	by KgNr-Ez
CPI	1	Yearly Consumer Price Index	by Year
ATX	2	Monthly median and 6 moths shifted in the past	by year and month
Socio-Demographic	5	crime, unemployed, average income, share of foreigners and average age	by year and ZIP code
Education	8	Different levels of education: including compulsory school, secondary school, apprenticeship among others	by year and district
Urban Core	32	variation of segment, junction, POI etc. statistical values based on a cell grid	by location
BKM	3	Area, BSR, DI - based on UC grid cell	by location and Year
Shortest path	15	POI: doctor, drugstore, kindergarten, museum, parks, police station, low priority public transport, schools, subway single point, subway multi point, touristic attractions, train station, university, playground, restaurant/bar.	by location; individual
Isochrone	21x3	Shortest path POI with some fine distinction. Low-priority public transport is distinct in bus and tram; restaurant and bars are separate features. Subway features are finer grained	by location; individual
Time Isochrone	6x3	3 Transaction and Crime features counts	by location; individual and by Year
ZIP	1	based on the 23 districts of Vienna	individual

Table S1: The table contains all features included in the final version of the dataset. Each entry contains the feature class, the number of features within that class, a brief description, and the basis on which they are linked to the target data point. This feature overview represents an updated and extended version of the dataset description originally presented in [1].

Appendix B: System Architecture and Workflow

The workflow of the study consists of seven main stages, from data acquisition to evaluation. The following list summarizes the process and illustrates how the dataset, model, and human-subject experiment are connected within the research design.

1. **Data Acquisition**
   - Combined real estate transaction records (2010–2022) with external data sources: socio-demographic indicators, education statistics, CPI, ATX, and spatial accessibility measures (e.g., shortest paths, isochrones).
   - Implemented a cadastral identification system to ensure building-level consistency and prevent duplicate entries.
2. **Data Preprocessing**
   - Duplicate entries were removed, harmonized attribute formats were harmonized, and consistency was ensured across all data sources.
   - Engineered a total of 165 features that capture structural, locational, and socio-economic characteristics, including:
     - 15 shortest-path accessibility measures,
     - 21 isochrone-based accessibility indicators, each calculated for three travel-time thresholds,
     - 6 temporal isochrone metrics, also calculated for three time intervals.
   - Applied one-hot encoding for categorical variables to ensure comparability across data types.
3. **Model Training and Validation**
   - Employed the XGBoost algorithm on transaction data from 201–2021.
   - Conducted randomized hyperparameter tuning with five-fold cross-validation.
   - Tested one-, four-, and five-year input intervals; the five-year model achieved the lowest mean absolute percentage error (MAPE) and was selected for further use.
   - Performance was assessed using independent 2022 transaction data.
4. **Model Testing**
   - The selected five-year model was retrained using data from 2018–2022 to generate final predictions.
   - Based on the validation error and the median growth rate between validation and first-year predictions, the model's expected error was approximately 15%.

- Data point collection as described in subsection Human Subject Experiments - Object selection process.
- Model prediction of selected 15 properties

5. **Experimental Material Preparation**
   - Designed and implemented all experimental materials in LimeSurvey, reviewed and approved by the TU Wien Research Ethics Committee.
   - Developed three structured survey components: demographic questionnaire, expertise questionnaire, and experimental task section.
   - Prepared and formatted all visual and contextual materials, including tables and maps (e.g., comparison transactions, square meter price time series, and socio-demographic context).
   - Ensured randomized condition order to mitigate learning effects and standardized the data presentation for all participants.
6. **Human-Subject Experiment**
   - Selected 15 representative properties (5 per condition) from the 2022 dataset.
   - Conducted a within-subject design with 13 professional appraisers across three conditions: (A) human-only, (B) model-only, and (C) hybrid (human + model).
   - Each participant estimated the price per square meter; workload and completion times were recorded using the NASA TLX questionnaire.
   - Short Debrief at the end of the sessions
7. **Evaluation and Analysis**

Appendix C: Questionnaire Summary

The following table S2 summarizes the questionnaire used in the human–subject experiment. It outlines the main sections, content focus, and question formats as implemented in the LimeSurvey platform. This structure ensures transparency of the experimental process and supports reproducibility.

Section	Content Overview	Question Type(s)
**Demographic Information**	Age, gender, highest educational level, employment status, and residential history in Vienna (current residence, past residence, years lived in Vienna).	Multiple choice, numeric entry
**Expertise Questionnaire**	Current and past employment in the real estate industry, role (agent or appraiser), years of experience, specialization by property type or market, familiarity with the Viennese market, experience in other markets, areas of expertise, and self-assessed competence in predicting new-build apartment prices in Vienna.	Multiple choice, Likert scale, free text
**Condition A – Limited Information**	Participants estimate the price per square meter based only on core property attributes (ZIP code, size, floor, penthouse status, date, and buyer class) without access to supporting data.	Numeric entry
**Condition B – Professional Context**	Additional professional data are provided, including comparable transactions, time series of square meter prices, and neighborhood information.	Numeric entry
**Condition C – Hybrid (Human + Model)**	Same as Condition B, supplemented with model-generated price suggestions and an indicated error margin (±15%).	Numeric entry
**Information Evaluation**	After each condition, participants rate the helpfulness of different information categories (object, neighborhood, socio-demographic, education, infrastructure, comparison, square meter price, own research, estimated price).	5-point Likert scale
**NASA TLX Workload Assessment**	After each condition, perceived workload assessment per condition (mental demand, physical demand, temporal demand, performance, effort, frustration).	Likert scale (1–5)

Table S2: Summary of the experimental questionnaire structure.

References

[1] Kmen, C., Navratil, G. & Giannopoulos, I. *Location, Location, Location: The Power of Neighborhoods for Apartment Price Predictions Based on Transaction Data. ISPRS International Journal of Geo-Information*, **13**(12), 425 (2024).
